# Ultrasound‐Activated Bifunctional Piezoelectric Hydrogel Dressings Promote Infected Wound Healing via Regulating Angiogenesis and Lymphangiogenesis

**DOI:** 10.1002/advs.202522152

**Published:** 2026-04-27

**Authors:** Xiang Li, Zhen Ding, Zhihua Liu, Hao Chen, Jianyang Shan, Yanxuan Shao, Yaling Yu, Gen Wen

**Affiliations:** ^1^ Department of Orthopedic Surgery Shanghai Sixth People's Hospital Affiliated to Shanghai Jiao Tong University School of Medicine Shanghai China; ^2^ College of Fisheries and Life Science Shanghai Ocean University Shanghai China; ^3^ Institute of Microsurgery on Extremities Shanghai Sixth People's Hospital Affiliated to Shanghai Jiao Tong University School of Medicine Shanghai China; ^4^ Department of Osteoporosis and Bone Disease Shanghai Clinical Research Center of Bone Disease Shanghai Sixth People's Hospital Affiliated to Shanghai Jiao Tong University School of Medicine Shanghai China; ^5^ College of Information Engineering Shanghai Maritime University Shanghai China

**Keywords:** antimicrobial capabilities, infected wound healing, lymphangiogenesis, piezoelectric hydrogel

## Abstract

Impaired wound healing resulting from bacterial infections represents a major clinical challenge, necessitating the development of wound dressings simultaneously possessing superior antimicrobial properties and excellent promoting wound healing ability. In this study, we designed a double network piezoelectric hydrogel AB‐Gel, leveraging ultrasound‐triggered piezoelectric‐catalyzed therapy, to enhance healing in infected wounds. Using acryloylglycine and gelatin as substrates, we embedded barium titanate nanoparticles within the hydrogel. The hydrogel exhibited excellent adhesion and reproducibility. Ultrasonic stimulation induced a synergistic effect combining acoustic activation and piezoelectric polarization, leading to enhanced reactive oxygen species (ROS) generation at the hydrogel interface, endowing the AB‐Gel hydrogel with strong antimicrobial capabilities. In vivo and in vitro experiments showed that the piezoelectric AB‐Gel significantly accelerated infected wound healing by regulating inflammatory response, promoting granulation formation, angiogenesis, and lymphangiogenesis, a process rarely reported during wound healing. Consequently, the piezoelectric AB‐Gel hydrogel orchestrates the infected, refractory wounds' microenvironment to facilitate wound healing and will be of high value in the treatment of infected wounds. As such, this piezoelectric hydrogel provides a promising, versatile option for clinical translation.

## Graphical abstract

This study developed a double‐network hydrogel with piezoelectric properties, exhibiting excellent antibacterial activity and promoting fibroblast migration, angiogenesis, and lymphangiogenesis through piezoelectric stimulation, thereby accelerating the healing of infected chronic wounds.

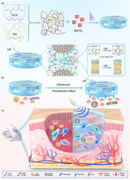

## 1 Introduction

Infectious chronic wounds represent one of the major global challenges for healthcare systems, imposing a substantial burden on individuals, families, and society at large [1]. Unlike normal wound healing, which follows a predictable sequence of phases (inflammation, proliferation, and remodeling), the healing of infectious chronic wounds is often unpredictable, characterized by non‐linear dynamics in which multiple phases coexist simultaneously, thereby rendering the process more complex and prolonged [2]. Moreover, these wounds are frequently associated with comorbidities such as diabetes [3], vascular dysfunction [4], and pressure‐related injuries [5], and tend to remain arrested in a persistent infection‐driven inflammatory stage, significantly delaying recovery. In conventional clinical practice, antibiotics are the primary strategy for controlling wound infections. However, the bacterial biofilms formed at wound sites secrete extracellular polysaccharide matrices [6, 7], which act as physical barriers that markedly hinder antibiotic penetration, leading to resistance levels increased by 10–1000 fold [8]. The recent emergence of multidrug‐resistant strains has further compromised the efficacy of antibiotics. In addition, infectious chronic wounds are characterized by hallmark pathological features, including elevated wound pH and exudates enriched with proteases, which accelerate granulation tissue senescence and edema. These factors collectively impair fibroblast function, disrupt collagen deposition, and delay centripetal wound contraction, ultimately impeding wound healing.

Among the emerging therapeutic strategies, sonodynamic therapy (SDT) has demonstrated unique advantages. SDT harnesses the cavitation effect of ultrasound to activate sonosensitizers, thereby converting acoustic energy into chemical energy and generating ROS, such as singlet oxygen and hydroxyl radicals, to achieve potent antibacterial activity [9]. Compared with photodynamic therapy (PDT), which is limited by a tissue penetration depth of only millimeters, ultrasound can penetrate several centimeters, conferring SDT superior therapeutic precision and deeper tissue reach [10]. The antibacterial efficacy of SDT primarily relies on ROS‐mediated damage to microbial lipids, proteins, and DNA [11]. The performance of SDT is largely determined by the properties of the sonosensitizer. Reported sonosensitizers include noble‐metal‐doped inorganic particles (e.g., TiO_2_, Cu_2_O), which exhibit high stability, facile synthesis, relatively low cost, and in some cases, additional photocatalytic activity that enables PDT/SDT synergism. Nevertheless, their limitations include restricted biocompatibility, poor dispersibility under physiological conditions, and ROS yields highly dependent on doping type and degree [12]. Alternatively, organic polymer‐based sonosensitizers (e.g., porphyrin‐based single‐atom catalysts) offer excellent biocompatibility, tunable chemical structures for improved targeting or solubility, and flexible design derived from natural or biomimetic scaffolds. However, they often suffer from limited chemical stability, susceptibility to structural degradation under prolonged ultrasound exposure, complex synthetic routes, higher costs, and uncertain in vivo clearance pathways [13]. Piezoelectric nanomaterials have emerged as particularly promising sonosensitizers, owing to their ability to generate internal electric fields under ultrasound stimulation via the piezoelectric effect. These fields enable efficient ROS generation without requiring exogenous molecular sonosensitizers, while the induced microcurrents or localized electric fields further enhance antibacterial activity through synergistic mechanisms. Importantly, biocompatibility and biodegradability remain essential prerequisites for wound‐healing applications. Barium titanate (BTO), with its intrinsic piezoelectric properties, has shown high ROS production under ultrasound, enabling direct bactericidal activity and biofilm penetration while overcoming antibiotic resistance. It has been widely explored in tissue engineering, particularly in composite scaffolds for hard tissue regeneration, where it exhibits remarkable potential [14, 15]. However, its application in cutaneous wound healing remains scarcely reported.

In contrast, other piezoelectric materials, such as poly(vinylidene fluoride) (PVDF), zinc oxide (ZnO), and poly(L‐lactic acid) (PLLA), have been extensively explored as hydrogel‐based wound dressings. These systems leverage various forms of mechanical stimuli, including low‐frequency deformation (e.g., bending, stretching, compression) and high‐frequency ultrasound vibration, to generate electrical signals, and have been fabricated as electrospun nanofibers, self‐powered nanogenerators, or composite hydrogels [16]. Recent studies have demonstrated that such piezoelectric hydrogels can effectively eliminate bacteria, modulate inflammation, promote angiogenesis, and enhance collagen remodeling. However, during wound healing, the potential of piezoelectric hydrogels to promote lymphangiogenesis—a critical process for resolving edema, clearing inflammatory mediators, and restoring tissue homeostasis‐ has never been specifically investigated [17].

**SCHEME 1:**
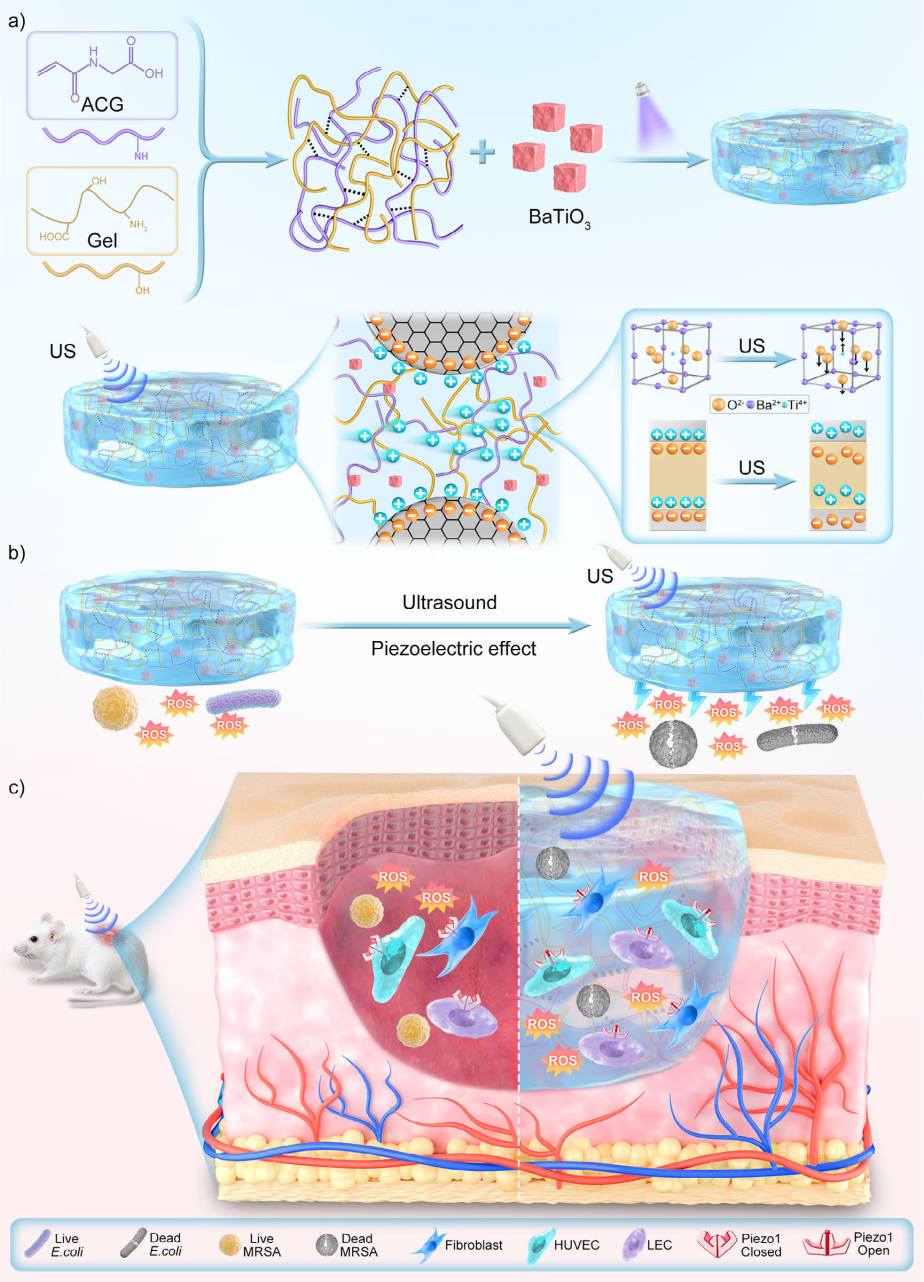
Schematic diagram of the preparation of AB‐Gel piezoelectric effect double network hydrogel and its mechanism in promoting the healing process of infectious wounds. (a) The synthesis of AB‐Gel hydrogel and its piezoelectric mechanism. (b) The antibacterial effect of AB‐Gel hydrogel, in which ultrasound‐mediated piezoelectric stimulation induces excessive ROS generation in the surrounding environment, thereby promoting bacterial death. (c) The mechanism by which AB‐Gel hydrogel promotes the healing of infected wounds.

In this study, we innovatively developed a composite double network hydrogel dressing, acryloylglycine‐BTO‐gelatin (AB‐Gel) (Scheme 1). Ultrasound (US) treatment activated the hydrogel dressing to generate an obvious piezoelectric effect. This hydrogel dressing possessed perfect mechanical properties, strong adhesion, and good self‐healing properties. Under the ultrasonic‐triggered acoustic stimulation and piezoelectric catalytic effect, the multifunctional hydrogel dressing exhibited outstanding antibacterial properties. The implantation of AB‐Gel in MRSA‐infected wounds showed elevated angiogenesis and accelerated wound healing. It is worth noting that the piezoelectric catalytic treatment with the adhesive AB‐Gel in the infected wounds was greatly associated with enhanced regeneration of lymphatic vessels during wound healing. This study provides a new perspective on designing multifunctional wound dressing materials, particularly valuable for treating chronic refractory wounds, especially those that are difficult to reach with traditional methods. Furthermore, we innovatively proposed that the piezoelectric induction of a double network hydrogel with BTO nanoparticles as piezoelectric materials can induce lymphatic generation and promote the healing of infected wounds.

## 2 Results and Discussion

### 2.1 Characterization of AB‐Gel Double Network Piezoelectric Hydrogel

**FIGURE 1:**
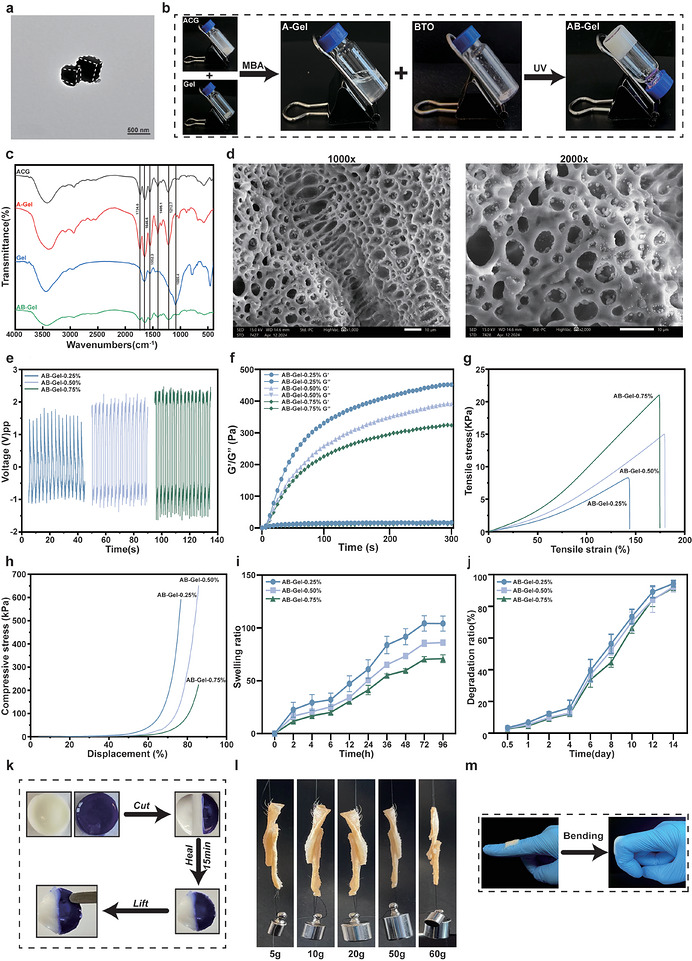
Characterization of the AB‐Gel. (a) SEM images of the BTO nanoparticles. Scale bar: 500 nm. (b) The preparation process of AB‐Gel hydrogel. After ACG and Gel are dissolved and crosslinked to form A‐Gel, then BTO nanoparticles are mixed with A‐Gel. (c) FTIR spectra of ACG, Gel, A‐Gel, and AB‐Gel hydrogel. (d) Representative SEM images of AB‐Gel‐0.50% hydrogels at different magnifications. (e) Open‐circuit voltage outputs of AB‐Gel hydrogels. (f) The rheological properties of AB‐Gel hydrogels. (g and h) Tensile stress‐strain curve and compressive stress‐strain curve of AB‐Gel hydrogels. (i,j) Swelling behavior and degradation curve of AB‐Gel hydrogels. (k) Test of the self‐healing properties of AB‐Gel hydrogel. (l) Pig skin viscosity test of AB‐Gel‐0.50% hydrogel. g represented gram. (m) Deformation ability test.

BTO nanoparticles are pivotal in biomedical applications, notably in targeted drug delivery, stem cell differentiation, and tissue regeneration, owing to their biocompatibility, electromechanical conversion, and high‐voltage charge coefficient [18]. BTO nanoparticles applied in this study were synthesized via a hydrothermal method [19], achieving a tetragonal phase crystal structure, which is exhibited in Figure 1a. As shown in Figure 1b, the double network piezoelectric hydrogel was fabricated in two steps. Acryloylglycine (ACG) and gelatin (Gel) were crosslinked via N, N'‐Methylenebisacrylamide (MBA) to form the ACG‐Gel (A‐Gel) network. Enhanced C═O and C─N vibrations at 1212 and 1646 cm^−^^1^, respectively, in the A‐Gel sample, demonstrating the formation of amide bonds (‐CONH‐) (Figure 1c). Furthermore, the broad O‐H and N‐H stretching peaks of Gel extended to low wavenumbers (up to 2500 cm^−^^1^), and this phenomenon was also observed in the A‐Gel sample with high intensity, suggesting strong hydrogen‐bonding interactions between the ACG and Gel molecules. Secondly, BTO was uniformly distributed within the hydrogel with a universal mixing strategy, which was subsequently exposed to ultraviolet (UV) irradiation, yielding the final ACG‐Gel‐BTO (AB‐Gel) hydrogel with a piezoelectric effect. To optimize the hydrogel performance, the AB‐Gel hydrogels with various BTO concentrations were synthesized (Figure S1a). The loose, porous microarchitectures of the AB‐Gel hydrogel depicted in Figure 1d featured a double network with alternating transverse and longitudinal orientations. Numerous BTO nanoparticles were doped within the interconnecting pore walls, providing the structural foundation for the piezoelectricity of the AB‐Gel hydrogel. As illustrated in Figure 1e, the open circuit voltage peak–peak (Vpp) of the AB‐Gel hydrogels increased from 2.9 to 4.1 V when the proportion of BTO increased from 0.25% to 0.75%, indicating the excellent electric energy output characteristics of the AB‐Gel hydrogels.

The irradiation of 365 nm ultraviolet (UV) on the AB‐Gel precursor solutions of different groups for 6 s, resulting in the storage modulus G′ superior to the loss modulus G″, thus successfully forming hydrogels, indicating that AB‐Gel hydrogels have the characteristic of rapid gelation (Figure 1f). The modulus decreased with the increase of BTO content (452 Pa for AB‐Gel‐0.25%, 392 Pa for AB‐Gel‐0.50%, and 324 Pa for AB‐Gel‐0.75%). To further quantify the mechanical properties of AB‐Gel hydrogel, we evaluated the stress‐strain behavior (Figure S1b). We found that the maximum tensile stress the hydrogel can withstand increases with the BTO concentration (Figure 1g). When the tensile strain of the hydrogel is between 10%‐40%, the tensile Young's modulus of different hydrogel groups were AB‐Gel‐0.25% (6.8 kPa), AB‐Gel‐0.50% (7.12 kPa), and AB‐Gel‐0.75% (12.9 kPa) (Figure 1 h).

The swelling kinetics of hydrogels under simulated physiological conditions (Figure 1i) exhibited a significant increase in the swelling rate over time, with the achievement of rapid swelling within 6 h, mainly attributed to their solid double network structure, and reaching equilibrium around 72 h. With the increase of BTO content, the equilibrium swelling ratio of the AB‐Gel decreased (Figure S1c). The main reason may be that BTO particles significantly increase the effective crosslinking density of the hydrogel network, and the combined effect of the space blocking effect caused by their spatial occupation and possible reduction of average hydrophilicity. Post the hydrogels reached swelling equilibrium, the degradation process began to accelerate obviously at day 4 (Figure 1j). Post day 4, the increase in BTO concentration reduced the degradation rate of the AB‐Gel hydrogels. As rigid reinforcing phases, BTO particles maintain the overall structural framework of the gel even when partial degradation occurs, effectively preventing rapid disintegration. At day 14, the degradation efficiency of all three hydrogels reached approximately 90%.

Although the AB‐Gel‐0.75% group exhibited superior piezoelectric and tensile properties compared with the AB‐Gel‐0.50% group, its excessively high BTO concentration resulted in poor light transmittance, leading to an overly rigid surface and relatively weaker gelation capacity in the interior compared with the other two groups (Figure S1d). Based upon the above results, the AB‐Gel‐0.50% possessed optimal physical performance, as it provides a favorable balance between enhancing the piezoelectric response and maintaining suitable mechanical properties. Importantly, this concentration prevents the decline in light transmittance and compressive modulus observed at higher loadings, which can be attributed to the so‐called ‘shell effect’.

The body's frequent movements can cause closed skin wounds to reopen. Therefore, an ideal wound hydrogel dressing must possess robust self‐healing and adhesive properties to ensure it remains in place during physical activity [20]. The self‐healing performance of the AB‐Gel hydrogel was investigated by placing two cut pieces of the material at room temperature, without any additional treatment. After 15 min, the healed hydrogel was able to be lifted and withstand self‐gravity, as shown in Figure 1k. To evaluate the adhesive properties, a shear test was conducted using porcine skin. As depicted in Figure 1l, the AB‐Gel‐0.50% was able to withstand a maximum weight of 60 g. Additionally, the hydrogel remained intact and adhered to the finger when the skin was bent (Figure 1m). While the adhesive property decreased in the AB‐Gel‐0.75% hydrogel. These results indicate that the hydrogel possesses favorable skin compatibility in terms of self‐healing and adhesion, making it a promising candidate for use as a wound hydrogel dressing.

### 2.2 Biocompatibility Assessment of AB‐Gel Hydrogel

**FIGURE 2:**
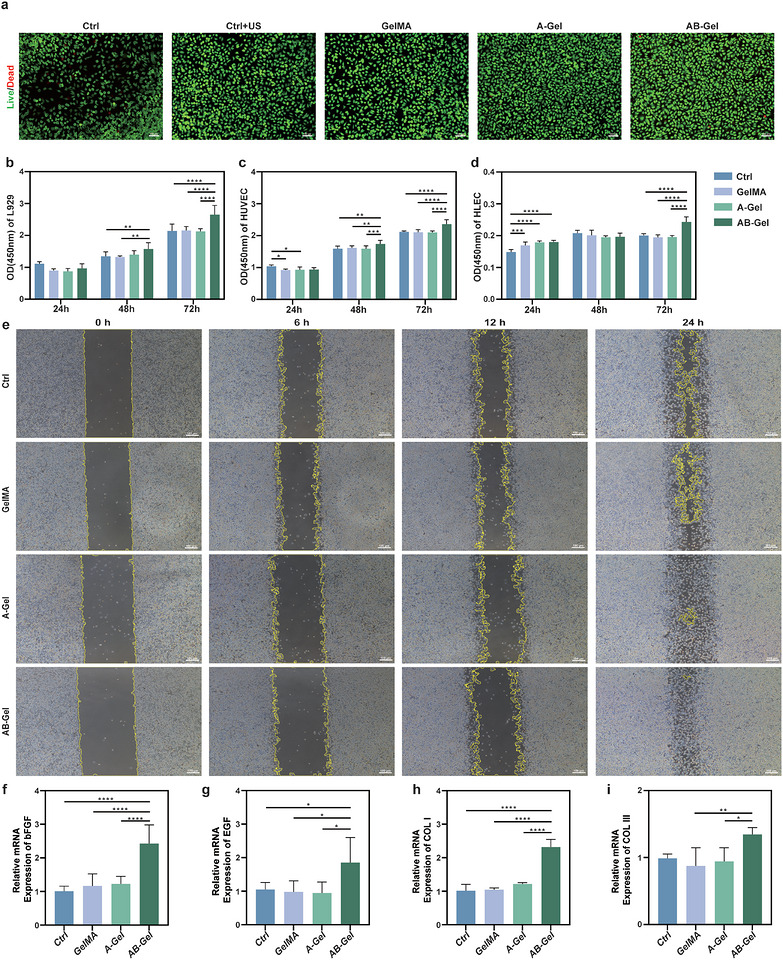
Biocompatibility of the hydrogels and their role in promoting cytokine secretion and cell migration. (a) Representative live/dead staining images of L929 cells after different treatments. Scale bar: 100 µm. (n = 3 independent biological replicates). Cell proliferation viability assay of L929 (b), HUVEC (c), and HLEC (d) co‐cultured with hydrogels (n = 3 independent biological replicates). (e) Representative images of the L929 cell migration assay at different times. Scale bar: 100 µm. (n = 3 independent biological replicates). Quantitative analysis of mRNA expression of (f) bFGF, (g) EGF, (h) COL‐I, and (i) COL‐III at day 3. Data are presented as mean±SD (n = 3 independent biological replicates). ^*^ *P* < 0.05, ^**^ *P* < 0.01, ^***^ *P* < 0.001, and ^****^ *P* < 0.0001.

Considering the potential risks of BTO nanoparticles to biological safety [21], the biocompatibility of AB‐Gel hydrogels is an important consideration for cell experiments and in vivo studies. In vitro cytotoxicity was evaluated according to ISO 10993–5 and ISO 10993‐12. Cultured on the AB‐Gel hydrogels, the survival rates of mouse fibroblasts were exceeding 90%, with no obvious difference among all three groups (Figure S2a,b). Compared with the control group, the Ctrl+US group did not exhibit significant thermal or mechanical damage to cells, and the AB‐Gel hydrogel also demonstrated comparable cell compatibility (Figure 2a and Figure S2b). To maintain experimental consistency, all groups underwent US treatment with identical parameters, including subsequent experiments. And the AB‐Gel hydrogel was superior to other groups in promoting fibroblast proliferation (Figure 2b). Besides, the AB‐Gel hydrogel promoted the proliferation of human umbilical vein endothelial cells (HUVEC) and human lymphatic endothelial cells (HLEC) (Figure 2c,d), indicating that AB‐Gel hydrogels may support the development of fibrous junctions and the angiogenesis and lymphangiogenesis during wound healing. The increase of BTO content within the AB‐Gel promoted the cellular proliferation rate (Figure S2c). Considering that the decreased adhesive property of the AB‐Gel‐0.75% hydrogel was unsuitable for the in vivo application, the AB‐Gel‐0.50% hydrogel was adopted later in this research. And the AB‐Gel hydrogel mentioned in the following research referred to the AB‐Gel‐0.50% hydrogel. To further evaluate the cytotoxicity of AB‐Gel hydrogel, an experimental condition medium containing 0.50% BTO nanoparticles was used. Compared with the blank control group, the cytotoxicity of the 0.50% BTO nanoparticle group was within the acceptable range (Figure S2d). In order to verify the biocompatibility of AB‐Gel hydrogel in vivo, a subcutaneous embedding experiment was carried out herein. 14 days later, no adverse reactions, including local ulceration, abnormal masses, and even animal deaths, were observed. Histological stainings revealed that no abnormal alterations, such as abnormal bleeding or inflammation outbreak in the skin tissue, heart, liver, spleen, lungs, and kidneys of the AB‐Gel hydrogel implanted group and the normal group (Figure S3). Although the short‐term biocompatibility of AB‐Gel was confirmed by subcutaneous implantation and histological analyses, the long‐term in vivo fate of inorganic BTO nanoparticles remains an important issue for clinical translation. BTO is a barium‐containing inorganic material with generally poor biodegradability. Following release from the degrading hydrogel during wound healing, BTO nanoparticles may accumulate in the liver, spleen, and kidneys, potentially inducing chronic inflammation or toxicity, as reported for other inorganic nanomaterials [22]. Therefore, systematic toxicological studies, including long‐term biodistribution, accumulation experiments, blood biochemical analyses, and histopathological assessments, are mandatory to clarify the core safety of this material system. This is a critical and indispensable step before any clinical application of this material system can be considered.

To assess the efficacy of AB‐Gel piezoelectric hydrogel in promoting cell migration, the scratch assay was employed on fibroblasts. As shown in Figure 2e and Figure S4, the AB‐Gel group exhibited the highest scratch closure rate, achieving 99.3% healing by 24 h, indicating that the AB‐Gel hydrogel can enhance fibroblast migration. While in the first 6 h, the cells in the AB‐Gel group and A‐Gel group migrated slowly, compared with the other two groups. This may be attributed to the slightly acidic pH of ACG, which could influence the cellular microenvironment. The fibroblasts' migration promoted by the AB‐Gel hydrogel was verified by the transwell assay (Figure S5a,c). In addition, the migratory capacity of HUVECs (Figure S5b,d) was greatly enhanced by the AB‐Gel hydrogel. During the interference of the AB‐Gel hydrogel, the EGF and bFGF expressions of the fibroblasts, which may enhance re‐epithelialization and accelerate wound repair [23, 24], were obviously up‐regulated (Figure 2f,g). Furthermore, collagen accumulation represents a key structural event during cutaneous wound repair, with collagen type I (COL‐I) and collagen type III (COL‐III) playing complementary roles in rebuilding the extracellular matrix and restoring tissue integrity [25]. Cells treated with AB‐Gel exhibited substantially higher transcript levels of COL‐I and COL‐III compared with the other groups (Figure 2h,i), suggesting an upregulation of collagen‐related gene expression associated with matrix remodeling. To sum up, the AB‐Gel hydrogel possessed excellent cytocompatibility.

### 2.3 AB‐Gel Piezoelectric Hydrogel Propels Vascular Regeneration and Lymphatic Vessel Regeneration In Vitro

**FIGURE 3:**
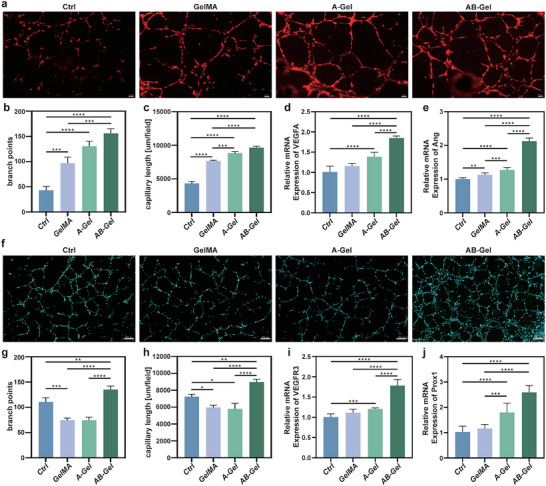
Effects of hydrogel on cell vascular generation and lymphatic tube formation in vitro. (a) Fluorescent images of HUVEC vascular generation under different co‐culture conditions. Scale bar:100 µm. (b and c) Quantitative analysis of vascular fluorescence expression (n = 3 independent biological replicates). (d and e) Expression of vascular generation‐related genes VEGFA and Ang (n = 3 independent biological replicates). (f) Fluorescent images of HLEC lymphatic tube formation under different co‐culture conditions. Scale bar: 100 µm. (g,h) Quantitative analysis of lymphatic fluorescence expression (n = 3 independent biological replicates). (i,j) Expression of lymphatic generation‐related genes VEGFR3 and Prox1. Data are presented as mean±SD (n = 3 independent biological replicates). ^*^ *P* < 0.05, ^**^ *P* < 0.01, ^***^ *P* < 0.001, and ^****^ *P* < 0.0001.

Angiogenesis and lymphangiogenesis play crucial roles in wound healing [26]. Recent studies have reported the piezoelectric effect's ability to promote endothelial cell activation [27]. Here, we investigated the in vitro angiogenesis and lymphangiogenesis potential of AB‐Gel hydrogel. Figure 3a depicted the tube formation data of HUVEC. Quantitative assessment showed 156 vascular junctions in the AB‐Gel group, substantially more than the control group and the GelMA group (Figure 3b). The total length of blood vessels induced by the AB‐Gel outperformed the control and GelMA groups (Figure 3c). The elevated expression of vascular endothelial growth factor A (VEGFA) and angiotensin (Ang) in the AB‐Gel group confirmed the pro‐angiogenic property of the AB‐Gel (Figure 3d,e).

We observed a similar trend in lymphangiogenesis in vitro, with the highest fluorescence intensity and lymphatic vessel regeneration effect in the AB‐Gel group, compared with the other groups (Figure 3f). Different from the decreased branch points and capillary length in the GelMA and A‐Gel groups, the AB‐Gel exhibited markedly increased lymphangiogenesis (Figure 3g,h). qRT‐PCR results indicated up‐regulation of lymphatic vessel formation‐related genes (VEGFR3 and Prox1) in the AB‐Gel group (Figure 3i,j). These findings suggest that the piezoelectric AB‐Gel hydrogel promotes both blood and lymphatic vessel formation.

### 2.4 Test of AB‐Gel Piezoelectric Hydrogel to Produce ROS at the Cellular Level

The piezoelectric effect of BTO nanoparticles can be leveraged to generate ROS under US stimulation [28, 29]. The intrinsic non‐centrosymmetric crystal structure of BTO gives rise to localized electric polarization under ultrasound stimulation, resulting in the formation of surface electric fields. These fields promote charge separation and facilitate redox reactions at the material interface, thereby enabling efficient generation of ROS through the piezoelectric effect. To confirm the US‐triggered piezoelectric catalytic activity of AB‐Gel hydrogel, the ROS generation of fibroblasts was assessed. Compared with the blank control, all the intervention groups up‐regulated the ROS generation, among which the ROS content in the US‐stimulated BTO group was the highest (Figure S6). Compared with the BTO group, the ROS production in the AB‐Gel group was slightly reduced, but was obviously superior to the GelMA and A‐Gel groups. It should be noted that the moderate antibacterial activity observed in the A‐Gel group under ultrasound is likely attributable to non‐specific effects, including the mildly acidic nature of acryloylglycine [30] and ultrasound‐induced mechanical perturbations [31], rather than piezoelectric catalysis. The results indicate that the elevated ROS observed in the AB‐Gel group should be interpreted as arising from the synergistic contribution of ultrasound stimulation and piezoelectric activation, rather than from intrinsic piezocatalysis alone. It should also be noted that ROS measurements performed at the cellular level represent a biologically integrated readout, reflecting not only material‐mediated ROS but also ultrasound‐induced physicochemical ROS and cell‐associated oxidative responses. Thus, the results indicate that ultrasound‐activated AB‐Gel establishes an enhanced oxidative microenvironment through the combined effects of acoustic stimulation and piezoelectric polarization. Importantly, ROS in wound healing is dose‐, time‐, and spatially dependent and may confer either beneficial signaling/antibacterial effects or detrimental oxidative injury when excessive or prolonged. While the antibacterial and pro‐regenerative benefits of ROS are well recognized, excessive or sustained ROS exposure can lead to lipid peroxidation, protein oxidation, DNA damage, and impaired tissue repair [32]. While the present study did not quantitatively define a therapeutic window for ROS generation or directly assess oxidative stress markers—representing areas for future investigation—the histological findings (Figure 5e and Figure S3) indirectly support that ROS levels remained within a physiologically tolerable range under the experimental conditions used. To further support the translational potential of the AB‐Gel system, future studies should systematically evaluate ROS dynamics across different healing stages, incorporate oxidative damage markers, and optimize ultrasound parameters to achieve controlled ROS output within a safe and effective therapeutic window. Additionally, long‐term biosafety assessments, including potential accumulation of BTO nanoparticles, will be essential prior to clinical translation.

### 2.5 Antibacterial Performance of AB‐Gel Piezoelectric Hydrogel In Vitro

**FIGURE 4:**
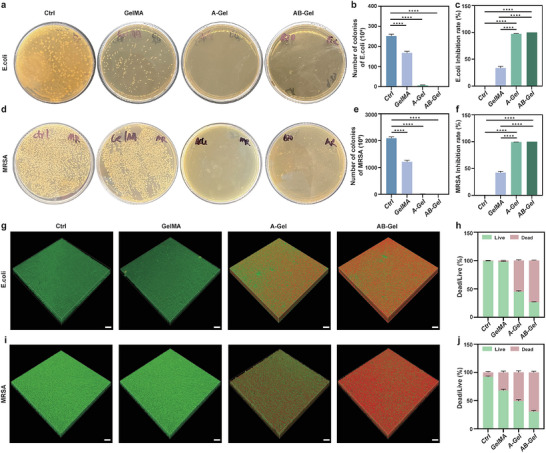
In vitro US‐triggered piezocatalytic antibacterial therapy of AB‐Gel against E.coli and MRSA. (a) Representative images of E.coli growth. (b and c) Quantitative analysis of E.coli colony number and inhibition rate (n = 3 independent biological replicates). (d) Representative images of MRSA growth. (e and f) Quantitative analysis of MRSA colony number and inhibition rate (n = 3 independent biological replicates). (g) Live/dead fluorescence staining images of E.coli in different culture environments. Scale bar: 100 µm. (h) Quantitative analysis of E.coli live/dead fluorescence staining. Data are presented as mean±SD (n = 3 independent biological replicates). (i) Live/dead fluorescence staining images of MRSA in different culture environments. Scale bar: 100 µm. (j) Quantitative analysis of MRSA live/dead fluorescence staining. Data are presented as mean±SD (n = 3 independent biological replicates). ^*^ *P* < 0.05, ^**^ *P* < 0.01, ^***^ *P* < 0.001, and ^****^ *P*< 0.0001.

Previous studies have shown that the bactericidal activity of ROS primarily arises from oxidative damage to membrane lipids, which compromises the structural integrity of bacterial membranes and ultimately leads to loss of viability [33, 34]. To evaluate the bactericidal efficacy of AB‐Gel hydrogel under US exposure, we conducted in vitro antimicrobial assays using smear plate and bacterial live/dead staining methods. To ensure consistent experimental conditions, all groups (Ctrl, GelMA, A‐Gel, AB‐Gel) were subjected to the same US treatment. As shown in the smear plate assays (Figure 4a,b), the *E. coli* colonies slightly decreased in the GelMA hydrogel. And the numbers in the A‐Gel group and the AB‐Gel group were the least, with the inhibition rate reaching 97.4% and 99.2%, respectively (Figure 4c). As one of the multidrug‐resistant bacteria, MRSA was greatly inhibited by the AB‐Gel hydrogel with the inhibition rate reaching 99.6% (Figure 4d–f). In addition, the bacterial live/dead staining assay conducted on four groups revealed similar trends in bacterial inhibition as observed in the coated plate experiments (Figure 4g–j). The percentage of dead bacteria reached as high as 69.41% for *E. coli* and 73.57% for MRSA in the AB‐Gel group. This disparity in results between the two methods can be attributed to differences in the initial bacterial inoculum and the duration of antimicrobial treatment. During the smear plate assay, bacteria were diluted to 10^6^ CFU/mL with a prolonged treatment duration of 12 h. In contrast, the bacterial concentration was diluted 10^3^ fold (10^9^ CFU/mL) during the live/dead staining process, requiring only 1 h of treatment time. Consequently, although the inhibitory results showed different outcomes, the gap between the A‐Gel and AB‐Gel groups widened as bacterial concentration increased and treatment time shortened. The enhanced antibacterial efficacy of AB‐Gel under ultrasound is attributed to the ROS generated through the combined effects of ultrasound stimulation and piezoelectric activation. Additionally, to better approximate biofilm‐associated infections in chronic wounds, a crystal violet staining‐based biofilm assay was performed (Figure S7). Representative images and image‐based quantification of the disrupted (cleared) area indicated that ultrasound‐activated AB‐Gel exhibited a more pronounced reduction in biofilm coverage/biomass than the control and GelMA groups. Notably, this assay provides a semi‐quantitative readout of total biofilm biomass/coverage and does not distinguish viable bacteria within biofilms or resolve biofilm architecture (e.g., thickness and 3D structure) and EPS matrix characteristics. Therefore, the current results support a biofilm‐interfering trend, while comprehensive biofilm‐specific validation remains necessary. To sum up, the AB‐Gel bi‐network hydrogel exhibited robust antibacterial properties, as evidenced by the experimental findings. Compared to traditional dressings, the localized controlled release of ROS avoids the risk of drug resistance and reduces toxicity to host cells. More importantly, the ultrasonic‐triggered piezoelectric hydrogel not only has antibacterial functions but also activates cellular responses through mechanical‐electrical signal conversion. A more reasonable interpretation for the antibacterial effect observed in the A‐Gel group is that it arises from the combined contributions of the mildly acidic ACG component (which may increase bacterial susceptibility and/or oxidative stress) and non‐specific ultrasound‐induced mechanical effects (such as acoustic cavitation and microstreaming). Notably, even in the absence of piezoelectric materials, these ultrasound‐related effects can still provide a baseline antibacterial action. This study did not systematically evaluate the long‐term dispersion stability of BTO nanoparticles within the hydrogel network under ultrasound stimulation or during hydrogel degradation. Potential aggregation of piezoelectric particles could be influenced by multiple factors, including (1) ultrasound‐induced mechanical disturbance and cavitation, (2) progressive degradation of the hydrogel network, and (3) localized electric fields or current effects generated during piezoelectric activation [35, 36]. Such aggregation could affect ROS generation uniformity and long‐term device performance. Future work should systematically assess particle distribution stability using techniques of degradation‐released particles and ultrasonic fatigue testing.

### 2.6 Test of AB‐Gel Piezoelectric Hydrogel on Promoting the Healing of Chronic Infection Wound

**FIGURE 5:**
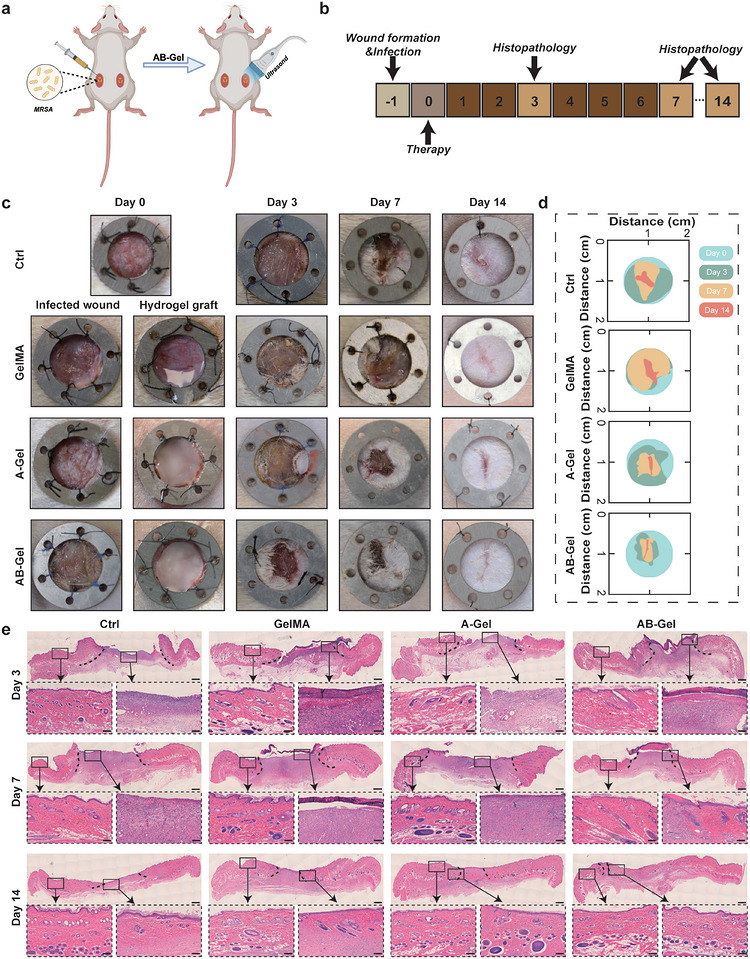
Evaluation of the effect of AB‐Gel hydrogel on the healing of infectious wounds in vivo. (a) Schematic diagram of AB‐Gel with US irradiation in the treatment of infectious wounds in rats. (b) Experimental timeline for animal wound model. (c) Macroscopic images of wound healing in rats on day 0, 3, 7, and 14. (d) Schematic images of wound closure trace during 14 days. (e) Representative H&E staining of skin lesions at different time points. Scale bar: 200 µm.Data are presented as mean±SD (n = 3 independent biological replicates).

All the above results indicated that AB‐Gel hydrogel may be applied as a potential dressing for infected wounds. A drug‐resistant MRSA‐infected rat full‐thickness defect model, which has been reported in the literature [37], was established to evaluate the efficacy of the AB‐Gel hydrogel in promoting wound healing. GelMA and A‐Gel were chosen as the control hydrogels. Considering that the piezoelectric effect of the AB‐Gel hydrogel was stimulated by US, an administration of 10 min of US irradiation was applied onto all groups post its graft on the wound (Figure 5a). The experimental timeline for the infected skin wound model was depicted in Figure 5b. As illustrated in Figure 5c,d, compared with the blank control wounds, all the hydrogel‐grafted wounds exhibited varying degrees of shrinkage. At all timepoints, the wound area of the AB‐Gel hydrogel group was significantly smaller than that of the other groups. After 3 days of treatment, the wound closure ratio of the AB‐Gel hydrogel group was approximately 20.5% higher than that of the A‐Gel group, 42.3% higher than that of the GelMA group, and 63% higher than that of the control group (Figure S8a), showing the best wound repair effect. After 7 days of treatment, among all the hydrogel groups, AB‐Gel exhibited the highest wound closure rate. While the wound closure ratio of the blank control group showed a rapid upward trend. At day 7, the wound was still in the transitional phase from inflammation to proliferation, and healing in the control group was mainly characterized by contractile repair. During the process of rat skin wound healing, myofibroblasts are highly active and tend to induce rapid surface contraction in the early stage through traction at the wound edges, thereby resulting in a relatively high closure ratio. As a hydrogel scaffold, GelMA may partially suppress the physical contraction of the wound, leading to a slower reduction in wound size compared with the control group. However, its primary role lies in providing structural support and modulating the microenvironment, thereby facilitating subsequent regeneration and tissue remodeling. On day 14, the wounds in the AB‐Gel hydrogel group were almost completely healed, with the wound closure ratio reaching 95.3%, and the wound closure ratios of the blank control, GelMA group, and A‐Gel group was 82.3%, 87.6%, and 90.4%, respectively (Figure S8a).

H&E staining revealed time‐dependent histological differences in wound healing among the groups (Figure 5e). On day 3, the control and GelMA groups exhibited extensive inflammatory cell infiltration with high cell density, evident tissue defects, and no epidermal formation. In contrast, the A‐Gel and AB‐Gel groups showed relatively reduced inflammatory responses, with the AB‐Gel group displaying the most compact wound margins and the least inflammatory infiltration. By day 7, the control group still exhibited pronounced inflammatory infiltration with limited granulation tissue formation and insufficient epidermal coverage. In the GelMA group, partial re‐epithelialization was observed, but large central defects remained, and dermal tissue appeared loosely organized. The A‐Gel group presented with abundant granulation tissue and reduced inflammatory cells, although epidermal regeneration was still incomplete. In the AB‐Gel group, inflammatory cells were markedly decreased, granulation tissue was denser, and relatively continuous neo‐epidermis had formed, with an increased epidermal thickness compared to the other groups. On day 14, the control group showed incomplete wound closure, residual inflammation, and a thin, discontinuous epidermis. The GelMA group exhibited uneven epidermal thickness and loosely arranged collagen fibers in the dermis. The A‐Gel group showed nearly complete epidermal coverage, but dermal appendages were limited. In contrast, the AB‐Gel group demonstrated complete wound closure with a continuous and thick neo‐epidermis, dense dermal structure, abundant collagen deposition, and the presence of follicle‐like structures, with minimal inflammatory cell infiltration. Overall, the AB‐Gel group exhibited the most favorable healing outcome, characterized by accelerated resolution of inflammation, enhanced epidermal regeneration, and improved dermal remodeling compared with the other groups. To further evaluate the wound healing effect of AB‐Gel, we quantitatively analyzed the number of hair follicles within the wound area (diameter 15 mm) in each group. At all timepoints, the AB‐Gel hydrogel group exhibited a superior amount of hair follicles, compared to the other three groups (Figures S8b and S9). On day 3, no obvious newly formed hair follicles were observed in any group by H&E staining. On day 7, the wounds remained in the transition phase from inflammation to proliferation, during which follicular regeneration had not yet entered an active stage. Consequently, no significant differences in follicle numbers were observed among the groups. The healing process in the control group was predominantly contraction‐driven. During rat skin wound repair, myofibroblasts are highly active and exert traction forces that pull the wound margins toward the center, resulting in a relatively higher closure ratio at this early stage. However, this mode of repair mainly reflects wound contraction rather than true tissue regeneration. In contrast, GelMA and A‐Gel, as hydrogel scaffolds, can partially inhibit such physical contraction, leading to a slower wound closure compared with the control group. Importantly, the role of GelMA lies in providing a supportive microenvironment that facilitates cell infiltration, angiogenesis, and extracellular matrix deposition, thereby favoring subsequent tissue regeneration and remodeling. At day 14, the increase in hair follicle numbers showed a trend positively correlated with wound closure in the control, GelMA, and A‐Gel groups, suggesting that the changes in follicle counts in these groups were largely attributable to wound shrinkage. In contrast, the AB‐Gel group exhibited a greater increase in follicle numbers than that expected from the trend of wound closure alone, indicating that the follicular increase in this group may originate from two sources: wound contraction and de novo folliculogenesis. These results indicate that the AB‐Gel hydrogel exhibits the best effect on infected wound healing. It should be noted that, although the Ctrl + US group was included in the in vitro experiments to minimize confounding from ultrasound exposure, the independent contribution of ultrasound itself to wound healing cannot be fully excluded in the in vivo setting. Previous studies have shown that therapeutic ultrasound, particularly low‐intensity pulsed ultrasound, may promote wound repair by modulating inflammation, enhancing angiogenesis, and facilitating tissue regeneration [38]. In addition, ultrasound‐assisted wound treatment has demonstrated beneficial clinical effects in chronic wounds [39]. Therefore, the in vivo therapeutic outcomes observed in this study should be interpreted as the result of ultrasound‐activated AB‐Gel treatment, rather than being attributed solely to the piezoelectric hydrogel itself.

### 2.7 AB‐Gel Hydrogel Promotes Angiogenesis and Lymphangiogenesis During Infected Wound Healing

**FIGURE 6:**
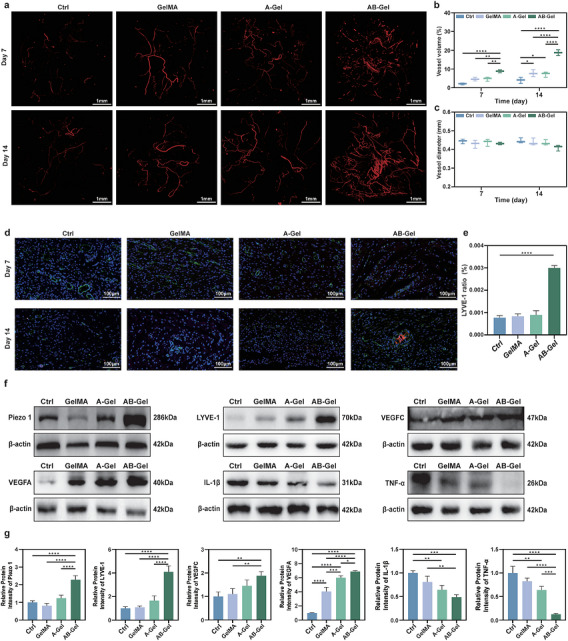
Analysis of angiogenesis and lymphangiogenesis during wound healing. (a) Representative images of Micro‐CT after vascular perfusion of skin tissue on day 7 and day 14. Scale bar: 1000 µm. (b and c) Quantitative analysis of vessel volume and vessel diameter at different time points (n = 3 independent biological replicates). (d) Representative images of CD31 (green) /LYVE‐1 (red) fluorescence staining of skin tissue on days 7 and 14. Scale bar: 100 µm. (e) Quantitative analysis of LYVE‐1–positive area, expressed as the percentage of LYVE‐1–positive area relative to the total tissue area. Data are presented as mean± SD (n = 3 independent biological replicates). (f) Western blot data of related proteins during wound healing. (g) Quantitative analysis of wound healing‐related protein expression. Data are presented as mean±SD (n = 3 independent biological replicates). ^*^ *P* < 0.05, ^**^ *P* < 0.01, ^***^ *P* < 0.001, and ^****^ *P* < 0.0001.

The development of a functional vascular network plays a pivotal role in skin wound repair by sustaining metabolic demands and coordinating intercellular signaling processes essential for tissue regeneration [40]. To assess the angiogenesis potential of AB‐Gel hydrogel, micro‐computed tomography (Micro‐CT) was performed following angiographic perfusion of rat skin. As illustrated in Figure 6a,b, Micro‐CT images exhibited layered vascular structures and increased vascular volume in AB‐Gel‐treated wounds at days 7 and 14 post‐operation, compared to the untreated and GelMA‐treated wounds. Quantitative analysis showed a decrease in the average vessel diameter of AB‐Gel over time, prompting vessel pruning during angiogenesis (Figure 6c). These results suggest that AB‐Gel hydrogel induces a highly functional angiogenic response during promoting infected wound healing. Furthermore, the cutaneous lymphatic system is increasingly recognized to facilitate wound healing and skin regeneration by regulating tissue inflammation, immunological responses, and fluid balance [17]. LYVE‐1, which has been identified as a specific marker for lymphatic vessels [41, 42], was stained on wounds from four animal groups. As illustrated in Figure 6d,e, there were almost no LEC in the control group at day 7, and until day 14 a few LYVE1‐positive LEC appeared around the newly formed blood vessels in the wound. The GelMA group and A‐Gel group exhibited slightly more LEC at the above two time points, while no significant lymphatic vessel formation was observed. By comparison, the AB‐Gel activated the highest number of LYVE1‐positive LEC and fostered significant lymphatic vessel formation around neovascularization at day 14 after surgery (Figure S10). This lymphangiogenesis induced by the piezoelectric AB‐Gel hydrogel supported the “self‐organization” hypothesis, although this hypothesis in existing literature mostly was reported in mouse tail wounds [17, 43].

To elucidate the detailed mechanism by which the piezoelectric AB‐Gel hydrogel facilitates wound healing, we assayed the expression of Piezo‐type mechanosensitive ion channel component 1 (Piezo1). Piezo1 is a mechanosensitive ion channel protein that transduces mechanical forces into electrochemical signals, thereby regulating cell membrane potential. This protein has been previously implicated in tissue regeneration and osteoarthritis treatment [44, 45, 46]. It has garnered significant attention due to its unique protein structure. It forms a tripartite propeller‐like ion channel with a central hat domain and extracellular domains, resembling three blades and consisting of 38 transmembrane helix units. These extracellular blade domains are connected to the central intracellular domain through three long bundle structures, enabling mechanical regulation mechanisms [47, 48]. As shown in Figure 6f, the Piezo1 expression was significantly higher in the AB‐Gel group compared to the other three groups. LYVE‐1 was expressed at the highest level in the AB‐Gel‐treated wounds, which was in line with the results of immunofluorescent staining (Figure 6d,e). Vascular endothelial growth factor C (VEGFC), a specific protein promoting lymphangiogenesis, was highly expressed in the AB‐Gel group. Pearson's correlation analysis revealed that Piezo1 expression was strongly and positively correlated with LYVE‐1 expression (r = 0.93, *p*<0.001), and moderately correlated with VEGFC expression (r = 0.76, *p*<0.01) (Figure S11). To provide pathway‐level validation for mechanosensitive involvement in lymphangiogenesis, we performed a Piezo1 inhibition/rescue assay during HLEC tube formation. Pharmacological inhibition of Piezo1 significantly reduced lymphatic tubulogenesis compared with the Ctrl group. Notably, ultrasound‐triggered AB‐Gel treatment partially restored tube formation under Piezo1 inhibition (Figure S12), suggesting that Piezo1‐related mechanotransduction contributes to lymphatic endothelial responses induced by ultrasound‐activated AB‐Gel. These data strengthen the functional linkage between Piezo1 activity and lymphatic endothelial tube formation, complementing our in vivo correlation analyses. Collectively, these findings indicate that ultrasound‐activated AB‐Gel is associated with enhanced angiogenic and lymphangiogenic responses, accompanied by elevated Piezo1/VEGFA/VEGFC signaling. Together with the Piezo1 inhibition/rescue results in vitro, our data support the involvement of Piezo1‐related mechanosensitive signaling in lymphatic endothelial responses, while recognizing that in vivo lymphangiogenesis is multifactorial and may also be influenced indirectly by improvements in the wound microenvironment [49, 50, 51]. Collectively, these results support an association between ultrasound‐activated AB‐Gel treatment and increased Piezo1/VEGFC signaling concomitant with enhanced LYVE‐1–positive lymphatic responses. Rather than establishing causality, our data suggest that Piezo1‐related mechanosensitive signaling may contribute to the lymphangiogenic phenotype observed in vivo. Although LYVE‐1 staining demonstrated enhanced lymphatic regeneration in the AB‐Gel‐treated wounds, the present quantitative analysis was mainly based on the extent of LYVE‐1–positive lymphatic structures. We acknowledge that functional evaluation of lymphangiogenesis should involve more than vessel area alone, and ideally include additional parameters such as lumen formation, vessel maturity, and network connectivity. Due to methodological limitations in the current study, these features were not quantitatively assessed. Therefore, our results should be interpreted as evidence that increased lymphatic regeneration is associated with improved infected wound healing, rather than as a complete functional characterization of regenerated lymphatic vessels. Future studies will focus on more comprehensive structural and functional analyses of lymphatic vessels to better define their role in the therapeutic effects of ultrasound‐activated AB‐Gel. These findings support a plausible cascade with the cascade mechanism of “mechanical force‐electrical signal‐biological effect”, highlighting the unique advantages of piezoelectric materials in tissue regeneration [52]. Additionally, there is a spatiotemporal synergy between vascular and lymphangiogenesis [53], that is, early vascularization provides nutritional and oxygen support, while later lymphatic network remodeling maintains tissue homeostasis by clearing inflammatory mediators and metabolic waste. This double‐system synergistic regeneration mechanism offers new insights for chronic wound treatment. Lymphangiogenesis during wound repair is multifactorial and can be indirectly promoted by reduced bacterial burden, improved perfusion, decreased inflammation, and enhanced tissue remodeling. Therefore, although our in vivo results demonstrate robust lymphatic enhancement and correlate with Piezo1/VEGFC expression, these data alone do not prove that lymphatic regeneration is a piezoelectric‐exclusive direct mechanism. Our Piezo1 inhibition assay provides supportive evidence at the cellular level; however, definitive causality and specificity in vivo will require additional loss‐of‐function and pathway‐blockade experiments (e.g., in vivo Piezo1 inhibition/knockdown and VEGFC/VEGFR3 blockade), as well as spatiotemporal mechanistic interrogation. For the infected chronic wounds, severe inflammatory responses induced by bacterial infections can impede the wound repair process [54]. TNF‐α and IL‐6, as key pro‐inflammatory cytokines, are known to remain highly expressed in infected wounds [55], which was confirmed by our WB results showing the highest levels in the blank control group. In contrast, hydrogel‐treated groups, especially the AB‐Gel hydrogel, exhibited a pronounced downregulation of both cytokines (Figure 6f). This suppression of excessive inflammation suggests that AB‐Gel hydrogel not only mitigates the severe inflammatory response but also creates a more favorable microenvironment for infected wound healing. The piezoelectric AB‐Gel hydrogel suppressed inflammation in the wound as evidenced by the quantitative analysis results, which were consistent with the macroscopic experimental findings (Figure 6g).

## 3 Conclusion

In summary, we successfully developed an ultrasonically triggered double network piezoelectric hydrogel. By crosslinking ACG, Gel, and BTO nanoparticles, the harvested AB‐Gel hydrogel generated an obvious piezoelectric effect under US stimulation. Owing to its mechanically robust and adhesive nature, along with intrinsic self‐healing behavior and good cytocompatibility, the hydrogel is suitable for use in skin wound environments. Ultrasonic activation enhances the antibacterial efficacy of AB‐Gel by synergistically promoting ROS generation through acoustic stimulation and piezoelectric activation. In vivo studies demonstrated that AB‐Gel hydrogel significantly enhanced wound healing efficacy in an infectious skin defect model, manifested as reduced inflammation, granulation tissue formation, collagen deposition, and improved wound healing rate. Notably, during the wound healing process, AB‐Gel hydrogel exhibits definitive direct or indirect promoting effects on angiogenesis and lymphangiogenesis, which play crucial roles in cutaneous wound healing. Taken together, these findings indicate that ultrasound‐activated AB‐Gel represents a promising strategy for promoting the repair of infected and refractory cutaneous wounds. By integrating piezoelectric responsiveness with a multifunctional hydrogel platform, this approach expands current design paradigms for wound dressings and offers potential advantages in addressing infections located beyond superficial tissue layers.

Despite the significant achievements of this study, several issues still require attention. First, optimizing ultrasonic parameters (such as frequency, intensity, duty cycle, and exposure duration) may further regulate ROS generation efficiency and cellular response. Second, the long‐term biosafety of BTO nanoparticles represents a critical barrier to clinical translation. Given their poor biodegradability and potential for organ accumulation, systematic long‐term toxicological studies, such as biodistribution, metabolic fate, blood biochemical analysis, and histopathological evaluation) are urgently required to fully assess their safety profile. In addition to long‐term biosafety, the dispersion stability of BTO nanoparticles within the hydrogel network under dynamic conditions also warrants careful consideration. In the present system, BTO nanoparticles are physically confined within the double‐network structure through polymer crosslinking and intermolecular interactions, which helps maintain their initial distribution. However, under prolonged ultrasound stimulation, acoustic cavitation and microstreaming effects may induce nanoparticle movement and collision, potentially leading to localized aggregation. Furthermore, as the hydrogel gradually degrades, the structural confinement weakens, which may result in the release of BTO nanoparticles and increase the risk of aggregation in the surrounding biological environment. Although SEM observations suggest a relatively uniform initial distribution of BTO nanoparticles, the present study did not directly evaluate their dispersion stability during long‐term ultrasound exposure or hydrogel degradation. Therefore, future studies should systematically investigate nanoparticle distribution and aggregation behavior using these techniques. In addition, surface modification strategies (e.g., polymer grafting or charge stabilization) may be explored to enhance the colloidal stability of BTO nanoparticles and improve the overall reliability of the system. Finally, the molecular mechanisms underlying lymphangiogenesis induced by piezoelectric stimulation require deeper mechanistic dissection, such as elucidating upstream mechanosensitive signaling (e.g., Piezo1‐mediated Ca^2+^ influx), downstream transcriptional regulators of VEGFC/PROX1, and potential crosstalk between lymphatic regeneration and peripheral nerve remodeling. Future research can explore the combined application of AB‐Gel with other therapies (such as stem cell therapy and growth factor release) or use spatial transcriptomics to reveal heterogeneous cellular responses under piezoelectric effects, thereby promoting its clinical translation.

## 4 Materials and methods

### 4.1 Synthesis of ACG Monomer

Typically, glycine (4.5 g, 60 mmol) was dissolved in 60 mL of potassium hydroxide solution (2 m). The resulting solution was cooled in an ice–water bath (0°C) for 10 min. Subsequently, acryloyl chloride (6 mL, 73.6 mmol) was added dropwise under continuous stirring using a dropping funnel. After completion of the addition, the reaction mixture was allowed to warm to room temperature and stirred for an additional 3 h. The reaction solution was then washed twice with diethyl ether to remove unreacted reagents and organic impurities. The aqueous phase was acidified to pH 2.0 using hydrochloric acid and extracted with ethyl acetate three times. The combined organic extracts were dried over anhydrous MgSO_4_, filtered, and concentrated under reduced pressure using a rotary evaporator to afford white solid products with a yield of 74%.

### 4.2 Preparation of BTO Nanoparticles

Weigh 10 mmol of tetrabutyl titanate (C_4_H_9_O)_4_Ti and place it in a Teflon cup with a 50 mL PTFE liner. Add 4 mL of anhydrous ethanol to dissolve it. After thoroughly mixing (C_4_H_9_O)_4_Ti with the anhydrous ethanol, quickly add 1.5 mL of ammonia solution (25‐28% NH_4_OH) and stir vigorously to hydrolyze (C_4_H_9_O)_4_Ti, forming a white sol. According to the predetermined barium‐to‐titanium ratio (Ba/Ti = 1.5), weigh 15 mmol of anhydrous barium hydroxide (Ba (OH)_2_·H_2_O) and stir to dissolve it in 5 mL of deionized water. Mix the dissolved barium hydroxide solution with the aforementioned white sol, stir thoroughly at room temperature, and then transfer it to the hydrothermal reactor. Before the closed hydrothermal reactor, 1.12 g triethanolamine was added to the mixture according to the requirements of experimental conditions (TEOA). The reaction time was set to 0.5 h to 48 h, with the reaction temperature ranging from 140°C to 200°C. After the reaction, the supernatant was transferred to an alkali solution container. The hydrothermal product was washed multiple times using glacial acetic acid, deionized water, and anhydrous ethanol (9000 rpm, 5 min) to thoroughly remove excess BaO and BaCO_3_. It was then dried in an oven at 80°C for 4 h, followed by grinding to obtain BaTiO_3_ powder. A small amount of BaTiO_3_ powder was placed in a crucible and calcined at 800°C to 1200°C for 4 h, and cooled in the furnace to obtain the heat‐treated BaTiO_3_ powder.

### 4.3 Preparation of AB‐Gel Piezoelectric Hydrogel

The hydrogel was prepared through a photo‐initiated free radical polymerization process. Specifically, 10% ACG and 2% Gel were used as the base materials, dissolved in 0.7 mL water and 0.3 mL 0.1% MBA. The mixture was thoroughly mixed by vigorous shaking to ensure complete dissolution. Subsequently, BTO nanoparticles of varying weights were added, and the mixture was ultrasonically oscillated at 37°C for 15 min to ensure thorough mixing. Finally, the mixture was cured using a 365 nm UV curing lamp. For cell experiments, the hydrogel was filtered and sterilized through a filter to ensure a sterile environment.

### 4.4 Hydrogel FTIR Determination

Fourier transform infrared spectroscopy (FTIR) was taken on a Nicolet iS20 (Thermo, USA). In the dry environment, the ATR attachment was placed in the optical path of the spectrometer to scan the air background. Then, the surfaces of different groups of hydrogel lyophilized block samples were tightly pressed against the crystal surface of the ATR attachment. The infrared spectra of the samples were collected with a resolution of 4 cm^−1^, 32 scans, and a test wave number range of 400–4000 cm^−1^.

### 4.5 SEM Morphology Analysis of AB‐Gel Piezoelectric Hydrogel

The hydrogels doped with BTO at different concentrations were lyophilized to prepare samples. The lyophilized samples were adhered to anti‐static tape and gold‐sprayed. Subsequently, SEM was used to photograph the microscopic morphology of the lyophilized AB‐Gel hydrogels. Scanning electron microscopy (SEM) images were acquired using a JCM‐7000F microscope (JEOL, Japan).

### 4.6 Test of Rheological Properties of AB‐Gel Piezoelectric Hydrogel

The rheological properties of AB‐Gel hydrogels with different BTO concentrations were measured by a rotational rheometer (Discovery HR2; TA Instruments, USA) with a cone‐and‐plate geometry (1°angle, 40 mm dia., 150 µm gap). A frequency sweep (0.2–10 Hz) was conducted on AB‐Gel hydrogels with series concentrations after UV crosslinking at 23°C. Another time sweep was also conducted on hydrogels for 300 s at 23°C with a frequency of 1 Hz, 1% strain. All the values were shown as the mean ± SD of three samples.

### 4.7 Test of Mechanical Properties of AB‐Gel Piezoelectric Hydrogel

The mechanical testing was conducted on an electromechanical universal testing machine (C43.104Y, MTS, China). The hydrogel was prepared into a rectangular prism measuring 1 cm × 3 cm in size and 2 mm in thickness. Using a clamp, both ends of the hydrogel were secured, and the robotic arm's tension was adjusted to zero. The hydrogel was then stretched uniformly at a speed of 0.03 mm/s until it ruptured. Similarly, the hydrogel was prepared into a cylinder with a height of 5 mm. After adjusting the robotic arm's stress to zero, the hydrogel was compressed uniformly at a speed of 0.03 mm/s, and the stress‐strain data of the hydrogel was recorded.

### 4.8 Hydrogel Piezoelectric Effect Assay

First, the piezoelectric device was prepared by freeze‐drying a series of AB‐Gel hydrogels and forming them into squares (1.5 cm × 1.5 cm). A 2 cm diameter circular copper foil was attached to the surface of the freeze‐dried sample as an electrode, and a 0.5 cm × 5 cm rectangular copper foil was extended from the copper foil to serve as a conductor. The aluminum foil backing was removed, and the cut copper foil (0.5 cm × 5 cm) was attached to the aluminum foil as the conductor. The device was then encapsulated and fixed with polyimide, resulting in a complete piezoelectric device. Then, using an electrostatic meter (from the American Keithley company), the voltage and current of a series of encapsulated AB‐Gel piezoelectric devices were measured. Metal pads (2 cm in diameter) were attached to both sides of the copper foil positions on the piezoelectric devices to concentrate stress and isolate induced charges. The wires of the piezoelectric devices were connected to the wires of the electrostatic meter. The piezoelectric devices were impacted with a force of 50 N at a speed of 10 cm/s and an acceleration of 800 cm/s^2^, with each impact lasting 1 s.

### 4.9 Swelling and Degradation Experiments

To measure the swelling property, the hydrogel samples were freeze‐dried using the FreeZone lyophilizer (Labconco, USA), and the initial weight (W_0_) of the freeze‐dried hydrogel samples was recorded. The samples were then immersed in a 37°C PBS solution (1×, PH = 7.4), with the PBS solution being changed every two days. This experiment lasted for 14 days until the hydrogel degradation reached over 90%. During the swelling study, the samples were weighed at specified intervals and recorded as (W_t_). The swelling rate (%) of the samples was calculated using the following formula:

$$\mathrm{Swelling}\;\mathrm{Ratio}\left(\%\right)=\left(W_{t}-W_{0}\right)/W0\times 100\%$$

For in vitro degradation, the hydrogel samples were washed and freeze‐dried. Then, the samples were immersed in PBS at 37°C, with the PBS solution (1×, PH = 7.4) being changed every two days. This experiment was continued until the weight of the hydrogel stopped increasing, and the duration of the experiment was 14 days. The samples were weighed on day 0 (W_0_) and at different specified days, with the weight recorded as (W_t_). The degradation rate (%) was calculated using the following formula:

$$\mathrm{Degradation}\;\mathrm{Ratio}\left(\%\right)=\left(W_{0}-W_{t}\right)/W0\times 100\%$$

### 4.10 Self‐Healing properties

The AB‐Gel hydrogel precursor solution was placed in a 24‐well cell culture plate to form the gel. Two pieces of hydrogel of equal size were selected for self‐healing experiments. One piece was stained with crystal violet to distinguish it from the other. Each hydrogel was divided into two halves, and the two pieces of hydrogel of different colors were placed together at room temperature and tightly adhered. The adhesion state of the two pieces of hydrogel was observed over time until they were fully adhered. The re‐adhered hydrogel was then lifted with tweezers, and if it could withstand its own tension, it was considered to have successfully healed.

### 4.11 Viscosity and Deformation Performance Test

The adhesion strength was evaluated through a tensile shear test. Specifically, fresh porcine skin was cut into 1 by 3 cm rectangles and soaked in PBS for later use. The hydrogel precursor solution was applied to the surface of the fresh porcine skin, then cured under UV light. Another piece of porcine skin of the same size was attached to the solution (with an adhesion area of 1 cm × 1 cm). Finally, different weights were suspended at the end of the porcine skin. If the corresponding weight could be lifted, it was considered successful. Until the corresponding maximum weight could not be lifted, the weight was recorded as the maximum tensile force that the hydrogel could withstand. The hydrogel was made into a size of 2 cm x 1 cm, and the hydrogel was made into rubber gloves under light. The finger was bent repeatedly to observe the change in the hydrogel with the bending of the finger.

### 4.12 Cell Culture and Viability

In vitro cytotoxicity was evaluated according to ISO 10993–5 and ISO 10993‐12. In this experiment, we utilized L929, HUVEC, and HLEC cells for cytological studies. L929 cells were cultured in DMEM medium with the supplement of 10% FBS and 1% P/S. HUVECs were cultured in endothelial cell culture medium obtained from Zhong Qiao Xin Zhou, China. HLEC cells were cultured in complete culture medium for human lymphatic endothelial cells obtained from Zhong Qiao Xin Zhou, China. The proliferation activity of cells (L929, HUVEC, and HLEC) was assessed using the CCK‐8 assay (Servicebio, China). In brief, at each time point, the CCK‐8 working solution was added to the culture medium and incubated for 2 h. The absorbance of the supernatant was then measured at 450 nm using a spectrophotometer. AB‐Gel hydrogels containing different concentrations of BTO were co‐cultured with L929 cells for 24 h, and the cells cultured on the hydrogel surface were fluorescently stained with a live/dead cell double staining kit (Beyotime, China). In brief, aspirate the excess medium around the hydrogel, rinse with PBS three times for 5 min each time. Add an appropriate volume of Calcein AM/PI detection solution according to the manufacturer's instructions, and incubate at 37°C in the dark for 30 min. After staining, place the hydrogel on a slide, cover it with a coverslip, and use a confocal microscope (Leica, Germany) to observe the cells within the hydrogel.

### 4.13 Cell Migration Ability

The L929 and HUVEC cells were inoculated into a 6‐well plate. When the cells reached 90% confluence, a 200 µL pipette tip was used to gently touch the plate and draw a straight line. The plate was then rinsed twice with PBS to remove any detached cells. The cells were then incubated in a serum‐free medium. Scars at different time points were recorded using an optical microscope (Nikon, Japan). Cell migration induced indirectly by hydrogels was evaluated using Transwell chambers (Corning, USA). L929 and HUVEC cells were seeded into the upper chambers at a density of 2 × 10^4^ cells per well. Culture medium containing different hydrogels was added to the lower chambers to serve as the chemoattractant. After incubation for 12 h, the non‐migrated cells remaining on the upper surface of the membranes were gently removed, and the migrated cells were washed with phosphate‐buffered saline (PBS) and fixed with 4% paraformaldehyde for 20 min. The cells were then permeabilized with 0.1% Triton X‐100 for 30 min, stained with crystal violet solution (Beyotime, China), and imaged under a light microscope.

### 4.14 Vascular and Lymphangiogenesis Experiments

HUVECs were co‐cultured with different hydrogels for 24 h, after which the cell suspension was collected and seeded onto angiogenesis slides. The slides were then covered with Matrigel (10 µL per well) to induce tubular formation and placed in a cell culture incubator for 10 h. To stain the tubular cells, the HUVEC cell culture medium was aspirated, and the cells were washed twice with pre‐warmed PBS. The cells were fixed with 4% polyformaldehyde for 10 min, followed by two washes with PBS, each lasting 10 min. The cells were then permeabilized with 0.5% Triton X‐100 for 5 min. After washing with PBS, the cells were incubated in the dark with the recommended concentration of phalloidin working solution (Beyotime, China) for 30 min. After washing three times with PBS, the nuclei were incubated with DAPI for 5 min. Fluorescence images were observed by DMI8 (Leica, Germany). Additionally, the evaluation of lymphatic tube formation was similar to angiogenesis experiments. In simple terms, calcein (Sigma–Aldrich, USA) was used to stain HLEC cells for lymphatic tube formation. After aspirating the HLEC cell culture medium, calcein was diluted 1:1000 with PBS. The cells were washed with PBS and then incubated in the diluted calcein solution in the dark for 30 min. Fluorescent images were observed using the optical microscope.

### 4.15 Piezo1 Inhibition and Rescue Assay During HLEC Tube Formation

To examine the relationship between Piezo1 activity and the tube‐forming capacity of HLECs, we performed Piezo1 inhibition and rescue experiments using the Piezo1 inhibitor GsMTx4 (MCE, USA) at a final concentration of 50 µg/mL. Briefly, HLECs were co‐cultured for 24 h under four conditions: Ctrl, Ctrl + GsMTx4, Ctrl + GsMTx4 + AB‐Gel (rescue), and Ctrl + AB‐Gel (rescue). After treatment, cells were collected as a single‐cell suspension and seeded onto angiogenesis slides. For Piezo1 inhibition, cells in the inhibitor‐treated groups were pre‐incubated with GsMTx4 at 37°C for 60 min, and the inhibitor was maintained throughout the entire tube formation assay. For tube formation, angiogenesis slides were pre‐coated with Matrigel (10 µL per well) to induce tubular network formation, followed by incubation at 37°C for 10 h. Fluorescent staining and imaging of lymphatic tubular structures were performed using the same procedure as described above.

### 4.16 RT‐PCR Assay

Quantitative real‐time polymerase chain reaction (RT‐PCR) was used to analyze the expression levels of genes related to healing (EGF, bFGF, COL‐I, and COL‐III), angiogenesis (Ang and VEGFA), and lymphangiogenesis (Prox1 and VEGFR3). Total RNA was extracted from L929 cells, HUVEC, and HLEC using the EZB RNA purification kit, and then quantified using a spectrophotometer. cDNA was synthesized using a color reverse transcription kit. qRT‐PCR was performed using SYBR Green Master Mix (EZB) according to the manufacturer's protocol. Gene expression levels were calculated using the 2^−ΔΔCt^ method, with primer sequences provided in Table S1.

### 4.17 Subcutaneous Embedding Experiment

Six‐week‐old SD rats were used for the subcutaneous embedding experiment of the AB‐Gel hydrogel. After anesthetizing the rats with isoflurane, a 1.5 cm longitudinal incision was made along both sides of the spine, in the back region of the rat, on a sterile operating table. The pre‐fabricated hydrogel was then implanted into the subcutaneous tissue, and the wound was sutured layer by layer. The wound was disinfected daily with iodine tincture. 14 days post‐surgery, the rat was dissected, and tissue sections were taken from the skin, heart, liver, spleen, lung, and kidney. H&E staining was used to observe the alterations in the harvested tissues and organs. The normal tissues and organs were set as the control group.

### 4.18 ROS Determine

In order to evaluate the changes of ROS in the surrounding environment promoted by the piezoelectric AB‐Gel hydrogel, ROS was measured on L929 cells under different culture conditions using the H_2_O_2_ content determination kit (Macklin, China), and absorbance was measured using a spectrophotometer at 415 nm wavelength.

### 4.19 In Vitro Antibacterial Test

Gram‐positive MRSA (ATCC 33591) and Gram‐negative *E. coli* (ATCC 25922) were selected for antibacterial experiments. In short, the bacterial suspension prepared at 500 µL (10^8^ CFU/mL) was added to 500 µL GelMA, A‐Gel, and AB‐Gel in a 24‐well cell plate, respectively, and a negative control group was set up. All groups were exposed to ultrasonic irradiation (1.5 W/cm^2^, 1 MHz) for 10 min, and the 24‐well plate was incubated in a 37°C incubator for 2 h. The bacterial suspension was separated from the hydrogel surface using sterile PBS and diluted 100 times (10^6^ CFU/mL). A 24 h plate coating was performed, and the bacterial survival status in each agarose plate was observed and recorded. Cultivate MRSA and *E. coli* until the logarithmic growth phase, then dilute the bacterial concentration 10^3^ fold (10^9^ CFU/mL) with normal saline. Place the bacterial lawn at the bottom of a 24‐well plate, add 300 µL of the diluted bacterial solution to the lawn, and incubate at 37°C for 24 h. Afterward, aspirate the bacterial solution from each well and incubate the lawn under the four conditions mentioned above for 1 h. All groups were also subjected to ultrasonic treatment for 10 min. Then, the bacterial slides were cleared twice using PBS solution for 5 min each time. The slides were stained with the bacterial viability staining kit (DMAO/PI) (Beyotime, China). In brief, an appropriate volume of the bacterial viability staining working solution was mixed with the bacterial slides and incubated in the dark at 37°C for 15 min. After staining, the staining effect was observed under the confocal microscope.

### 4.20 Biological Membrane Disruption Experiment

*E. coli* and MRSA suspensions (10^6^ CFU/mL) were inoculated into 24‐well cell plates and incubated for 72 h to allow biofilm formation on the well surfaces. After confirming biofilm development, the supernatant was gently removed, and 500 µL of GelMA, A‐Gel, or AB‐Gel was added to the respective wells, with a negative control group included. Following incubation at 37°C for 2 h, the treatments were aspirated, and the wells were washed with PBS. The biofilms were then fixed with anhydrous methanol at room temperature for 15 min, air‐dried after methanol removal, and stained with crystal violet for 20 min. Excess dye was removed by washing twice with PBS, and representative images were acquired. Biofilm disruption was semi‐quantified by measuring the cleared (disrupted) area from crystal violet images using Image J with a consistent threshold across groups. Crystal violet staining provides a semi‐quantitative readout of total biofilm biomass/coverage and does not distinguish viable from non‐viable bacteria within the biofilm.

### 4.21 Infected Wound Model In Vivo

The ethics of animal experimentation were approved by the Animal Ethics Committee of Shanghai Chengxi Biotechnology Co., Ltd. (CX052405093). Sixty 6‐week‐old SD rats were selected for the creation of infectious wound models. In brief, after anesthetizing and shaving, a 15 mm diameter circular full‐thickness skin incision was made on both sides of the back using a skin loop drill. Then, a 30 µL suspension of MRSA (10^6^ CFU/mL) was injected into the defect site to induce infection. Twenty‐four h later (recorded as day 0), all rats with successfully established infectious wound models were randomly divided into four groups. (Ctrl, GelMA, A‐Gel, and AB‐Gel). Randomization was conducted using a computer‐generated random number table, ensuring unbiased allocation of animals to each treatment group. Each animal had an equal probability of being assigned to any group. Different interventions were applied to each wound. All groups received ultrasonic irradiation for 10 min (1.5 W/cm^2^, 1 MHz). To monitor the healing process of infected wounds, photos were taken on days 0, 3, 7, and 14. The wound closure rate was calculated using the following formula:

$$\begin{array}{cc} \mathrm{Wound}\;\mathrm{closure}\left(\%\right) & =[wound\;area\;on(day\;0-a\;specific\;day)]\\ & \;/\left(\mathrm{wound}\;\mathrm{area}\;\mathrm{on}\;\mathrm{day}\;0\right)\times 100 \end{array}$$

### 4.22 Histological Analysis

Skin tissues were harvested, fixed in 4% paraformaldehyde for 48 h, and embedded in paraffin. 5 µm thick sections were cut along the longitudinal axis of the specimen for H&E staining.

### 4.23 Angiography and Micro‐CT Scanning

To evaluate vascular regeneration in the cutaneous wound healing process, experimental rats were perfused with Microfil (Microfil MV‐122, Flow Tech, USA) at days 7 and 14 post‐surgery. The perfusion process was conducted according to the previous studies [56]. Shortly after anesthesia with 4% chloral hydrate, the thoracic cavity was opened, and the infusion needle was placed into the left ventricle. Heparinized normal saline, 10% formalin, and 10 mL mixed Microfil solution were perfused successively. After the contrast agent was fully polymerized, the skin of the wound healing area, approximately 2 cm × 2 cm, was harvested and underwent Micro‐CT scanning (SKYSCAN 1176, Bruker, Kontich, Belgium) to quantify the vascular regeneration in the wound healing area. Then, 3D reconstructions were generated with the application of the CTVol software (Skyscan Company). Parameter of the vascular regeneration, including vessel volume/tissue volume (VV/TV) was analyzed using the CTAn software (Skyscan Company).

### 4.24 Immunofluorescence Staining

The tissue sections were blocked in PBS supplemented with 10% horse serum and 0.3% Triton X‐100 for 1 h at room temperature and then incubated overnight at 4°C with primary antibodies against CD31 (GB120005, Servicebio, China) and LYVE‐1 (NB600‐1008, Novus, USA). After washing, the sections were incubated with appropriate fluorophore‐conjugated secondary antibodies and counterstained with DAPI. Fluorescence images were acquired using a Leica DMI6 (Leica, Germany) fluorescence microscope under identical exposure settings for all groups.

Quantitative analysis of LYVE‐1 expression was performed using ImageJ software. Briefly, regions of interest (ROI) covering the wound area were selected, and LYVE‐1–positive signals were identified by applying a consistent threshold across all samples. The proportion of LYVE‐1 fluorescence was calculated as the percentage of LYVE‐1–positive area relative to the total tissue area within the ROI. At least three sections from each sample and three independent samples per group were analyzed.

$$\mathrm{LYVE}-1\;\mathrm{positive}\;\mathrm{area}\left(\%\right)=A_{\mathrm{LYVE}-1\;\mathrm{positive}}/A_{\mathrm{total}\;\mathrm{tissue}}\times 100\%$$

where A _LYVE‐1 positive_ represents the area of LYVE‐1‐positive fluorescence signal after thresholding, and A _total tissue_ represents the total tissue area within the same ROI.

### 4.25 Western Blotting

Skin tissue lysates from experimental animals were diluted with loading buffer and heated at 95°C for 6 min. Protein extracts were electrophoresed by 10% SDS‐PAGE and blotted onto the PVDF membranes (Millipore, Billerica, MA). Thereafter, the membranes were blocked in 5% non‐fat milk for 2 h and incubated with primary antibodies overnight at 4°C. Next, the PVDF membranes were incubated with peroxidase‐conjugated secondary antibodies for 1 h at room temperature. After sufficient rinsing with TBST, chemiluminescent signals were developed using the enhanced chemiluminescent reagents and detected using the Tanon Imaging System (Tanon‐4600). The primary antibodies involved were as follows: anti‐Piezo1 antibody (1:300, Proteintech, China), anti‐LYVE‐1 antibody (1:300, Proteintech, China), anti‐VEGFC antibody (1:300, Proteintech, China), anti‐VEGFA antibody (1:300, Proteintech, China), anti‐IL‐1β antibody (1:300, Proteintech, China), and anti‐TNF‐α antibody (1:300, Proteintech, China). Protein expression levels were quantified by densitometric analysis and normalized to β‐actin as the internal loading control.

### 4.26 Statistical Analysis

The data were representative of greater than three independent experiments. Statistical analysis was conducted by Prism 10.0. Statistical differences were determined by One‐way ANOVA and Tukey's multiple comparison test. All quantitative data are presented as mean ± standard deviation (SD). Unless otherwise stated, n represents the number of independent biological replicates. The differences were regarded as statistically significant with ^*^ *P* < 0.05, ^**^ *P* < 0.01, ^***^ *P* < 0.001, and ^****^ *P* < 0.0001.

## Author Contributions

Xiang Li: Writing – review & editing, Writing – original draft, Visualization, Validation, Methodology, Investigation, Formal analysis, Data curation, Conceptualization. Zhen Ding: Visualization, Validation, Methodology, Funding acquisition. Zhihua Liu: Visualization, Validation, Methodology, Investigation. Hao Chen: Visualization, Validation, Methodology. Jianyang Shan: Visualization, Validation, Methodology, Investigation. Yanxuan Shao: Validation, Supervision, Resources, Funding acquisition. Yaling Yu: Writing – review & editing, Validation, Supervision, Resources, Methodology, Funding acquisition, Conceptualization. Gen Wen: Validation, Supervision, Resources, Project administration, Methodology, Funding acquisition, Conceptualization.

## Conflicts of Interest

The authors declare no conflict of interest.

## Supporting information




**Supporting File**: advs75447‐sup‐0001‐SuppMat.docx.

## Data Availability

Research data are not shared.
