# Supplementary material for: Ultrasound‐Activated Bifunctional Piezoelectric Hydrogel Dressings Promote Infected Wound Healing via Regulating Angiogenesis and Lymphangiogenesis
Source: Adv Sci (Weinh). 2026 Apr 27;13(41):e22152. doi: 10.1002/advs.202522152 (PMC13335527; doi:10.1002/advs.202522152)
Supplement: Supplementary file 1 — Supporting File: advs75447‐sup‐0001‐SuppMat.docx. [file ADVS-13-e22152-s001.docx]

Supporting Information

**Ultrasound-Activated Bifunctional Piezoelectric Hydrogel Dressings Promote Infected Wound Healing Via Regulating Angiogenesis and Lymphangiogenesis**


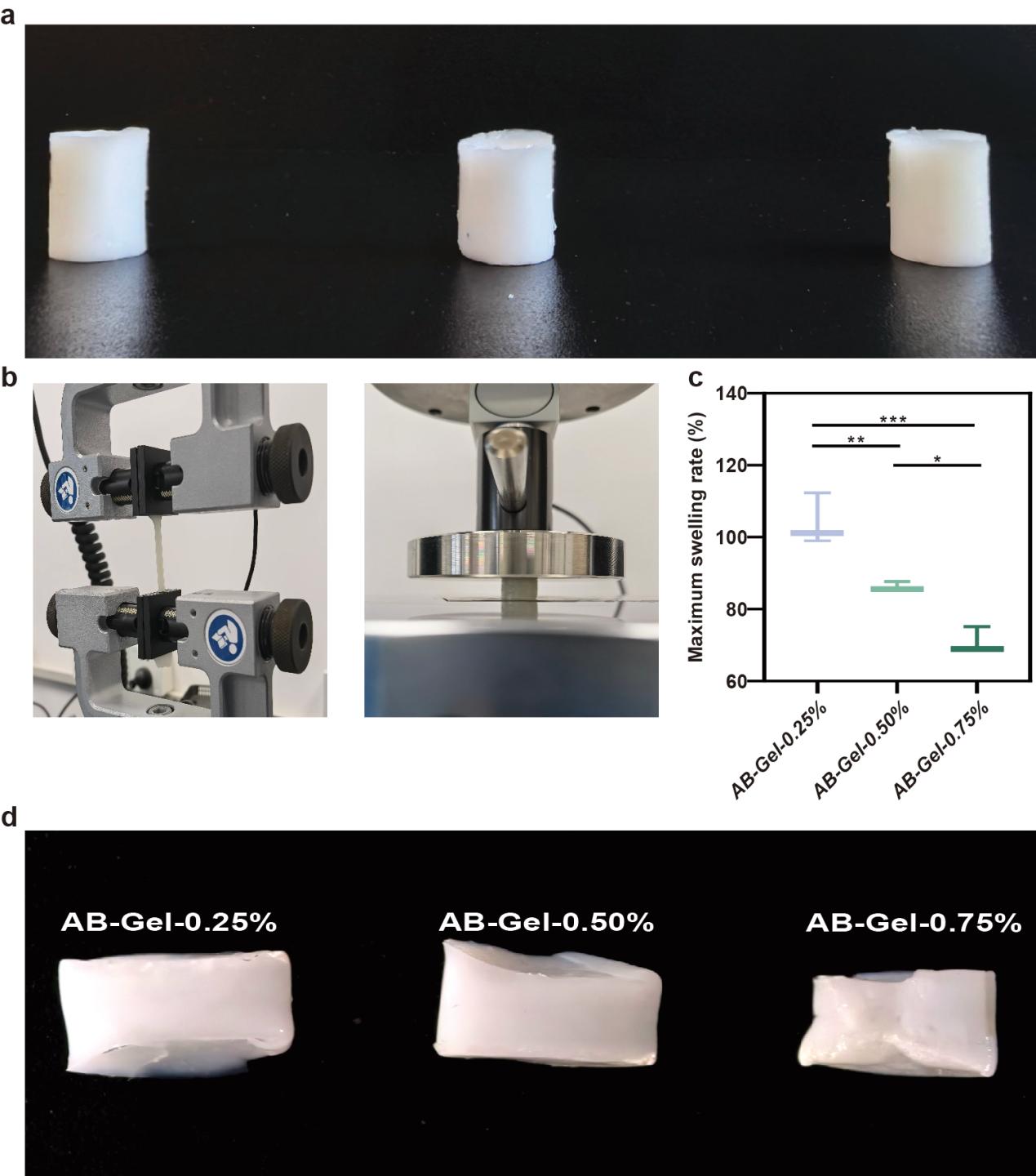


**Figure. S1. Characterizations of AB-Gel.** (a) Macroscopic images of AB-Gel hydrogels. AB-Gel-0.25% (left), AB-Gel-0.50% (middle) and AB-Gel-0.75% (right). (b) Mechanical test of AB-Gel hydrogel. Left panel represented tensile test and right panel represented compression test. (c) Quantitative analysis of the maximum equilibrium swelling ratio of AB-Gel hydrogels across different groups. (d) Cross-sectional appearance of AB-Gel hydrogels with three different concentrations of BTO. Data are presented as mean±SD (n=3 independent biological replicates). **P* < 0.05, ** *P* < 0.01, *** *P* < 0.001, and *****P* < 0.0001.


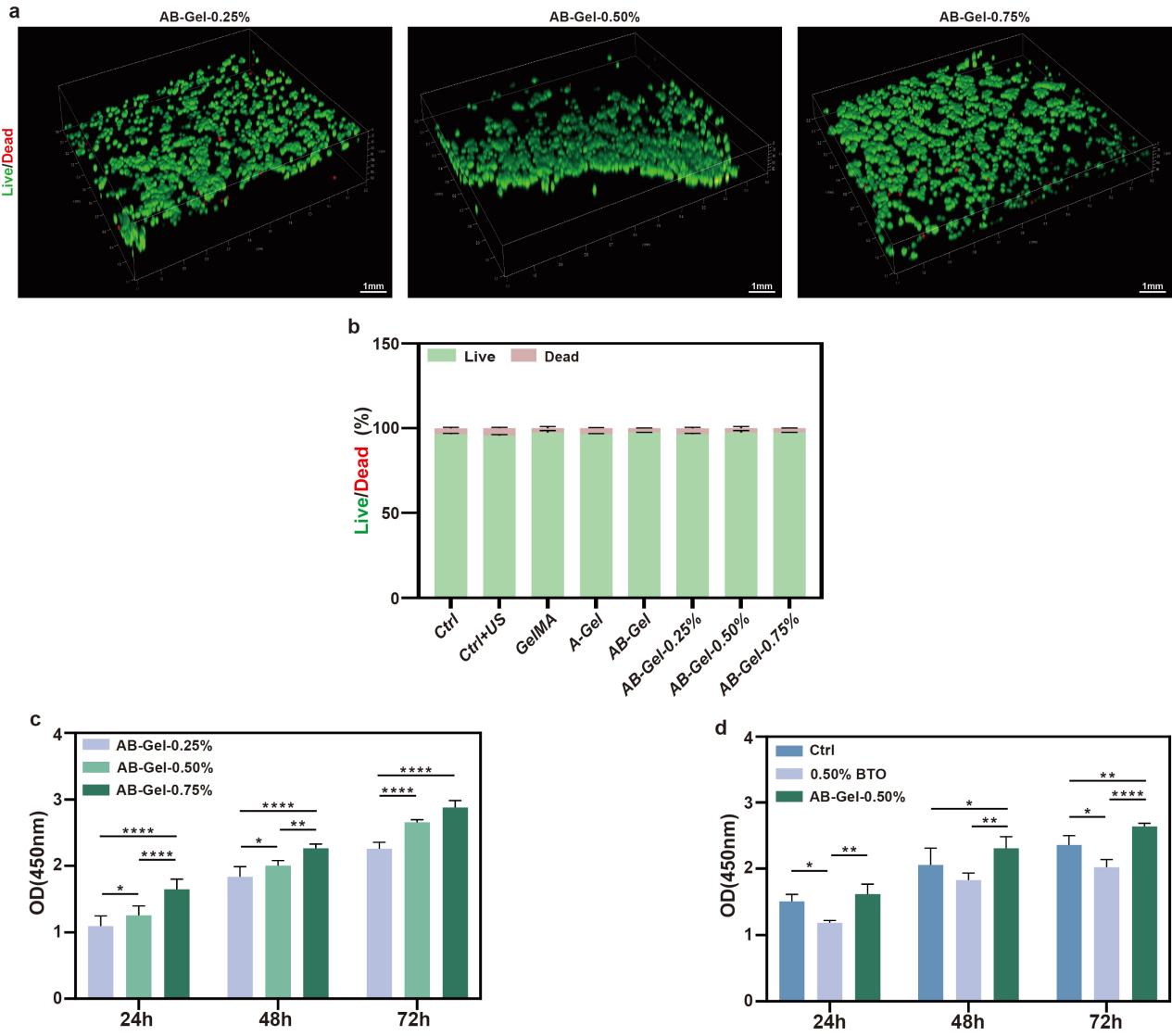


**Figure. S2. Cytotoxicity test.** (a) Representative images of live/dead staining of L929 cells. Scale bar: 1000 μm. (b) Quantitative analysis of live/dead cell staining in different experimental groups and hydrogels with three distinct BTO concentrations. (c) CCK-8 statistical results of all AB-Gel hydrogels. Data are presented as mean±SD (n=3 independent biological replicates). (d) Comparison of CCK-8 results between BTO nanoparticles and AB-Gel hydrogel. Data are presented as mean±SD (n=3 independent biological replicates). **P* < 0.05, ** *P* < 0.01, *** *P* < 0.001, and *****P* < 0.0001.


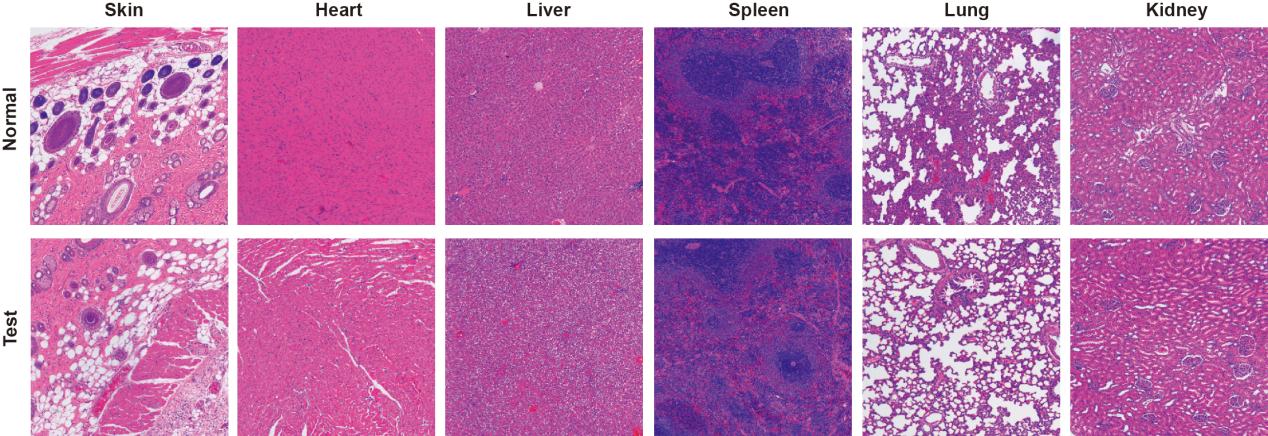


**Figure. S3. H&E staining of skin, heart, liver, spleen, lung and kidney in AB-Gel hydrogel (Test) group and Normal group.** Scale bar: 100 μm **(n=3 independent biological replicates)**.


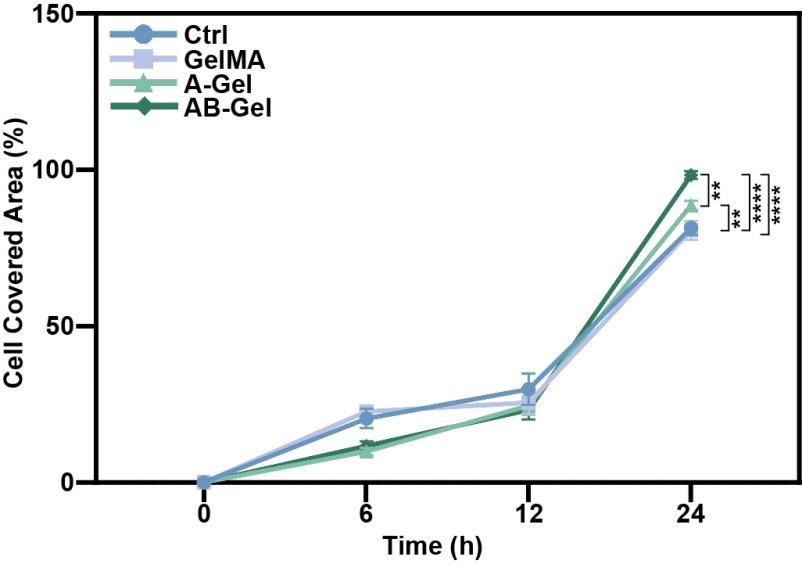


**Figure. S4. Quantitative analysis of cell scratch migration.** Enhanced cell migration in the AB-Gel group compared with the other three groups after 24 hours. Data are presented as mean±SD (n=3 independent biological replicates). **P* < 0.05, ** *P* < 0.01, *** *P* < 0.001, and *****P* < 0.0001.


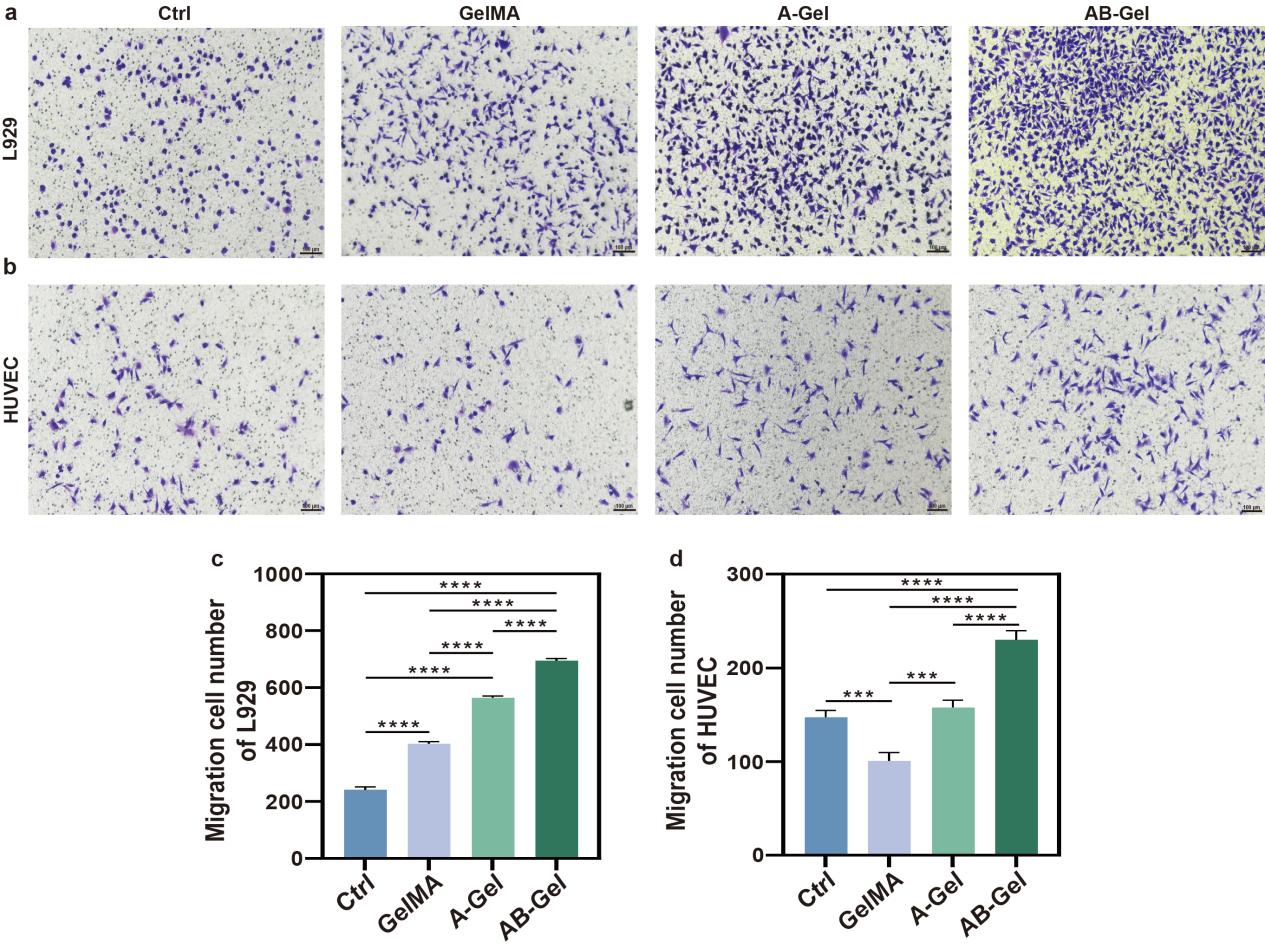


**Figure. S5. Transwell assay of L929 and HUVEC.** (a) Representative images of crystal violet staining of L929 cells. Scale bar: 100 μm. (b) Representative images of crystal violet staining of HUVEC. Scale bar: 100 μm. (c) Quantitative analysis of L929 cells migration. Data are presented as mean±SD (n=3 independent biological replicates). (d) Quantitative analysis of HUVEC migration. Data are presented as mean±SD (n=3 independent biological replicates). **P* < 0.05, ** *P* < 0.01, *** *P* < 0.001, and *****P* < 0.0001.


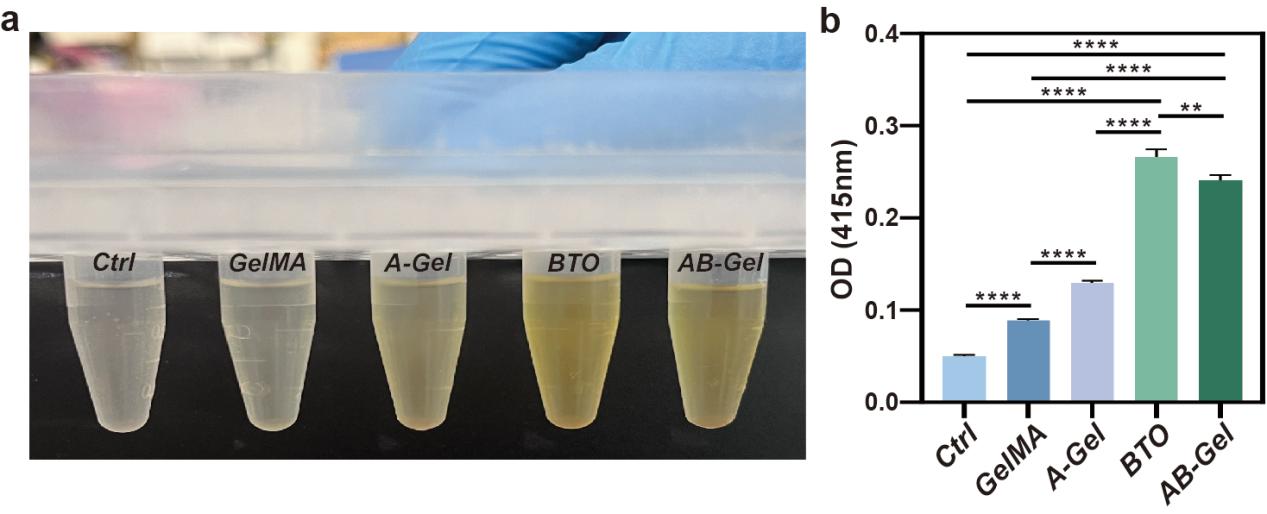


**Figure. S6. Cell-associated ROS levels in L929 fibroblasts under different treatment conditions.** ROS signals represent the cellular oxidative microenvironment in the presence of different hydrogels, rather than the intrinsic ROS yield of the materials alone. (a) Representative macroscopic images of ROS generation. (b) Quantitative analysis of ROS levels. Data are presented as mean±SD (n=3 independent biological replicates). **P* < 0.05, ** *P* < 0.01, *** *P* < 0.001, and *****P* < 0.0001.

**
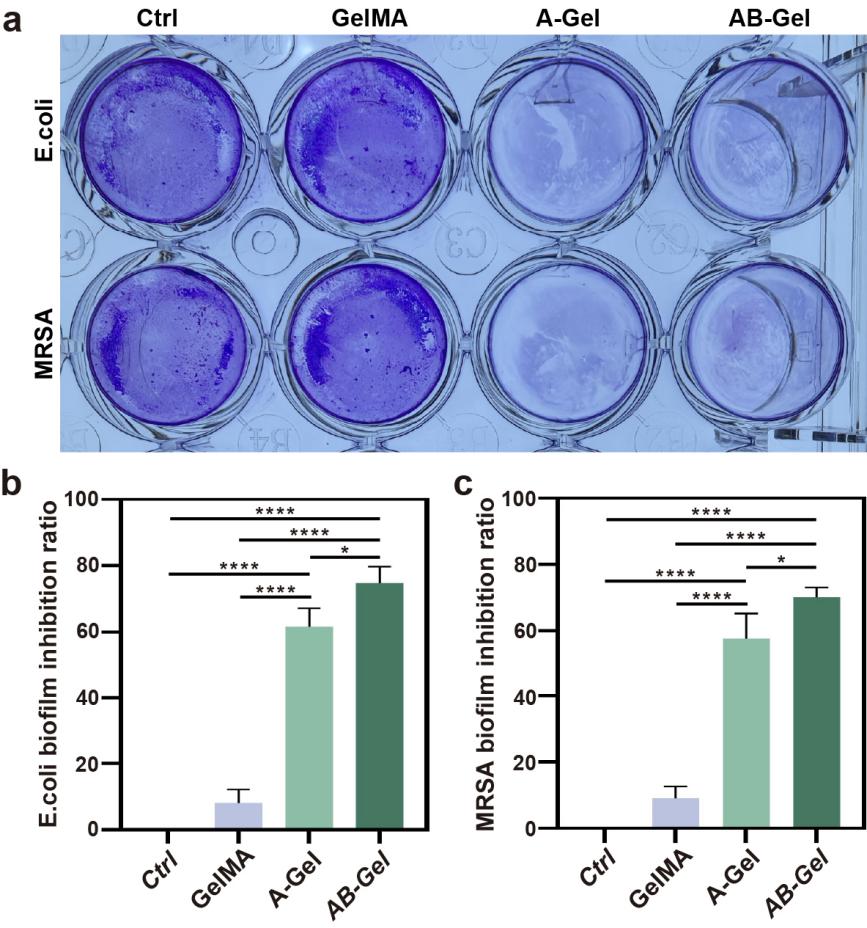
**

**Figure. S7. Biofilm disruption assay against *E. coli* and MRSA in different experimental groups.** (a) Representative images of bacterial biofilm disruption experiments. (b) Semi-quantitative analysis of *E. coli* biofilm inhibition ratio. (c) Semi-quantitative analysis of *MRSA* biofilm inhibition ratio. (n=3 independent biological replicates). **P* < 0.05, ** *P* < 0.01, *** *P* < 0.001, and *****P* < 0.0001.


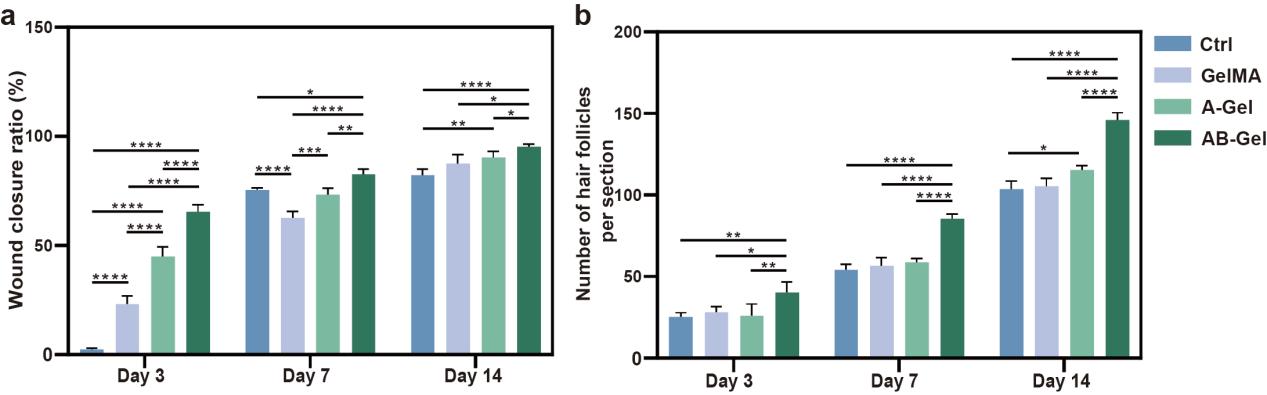


**Figure. S8.** (a) Quantitative analysis of wound healing rate. (b) Quantitative analysis of the number of hair follicles in the wound area of H&E staining. Data are presented as mean±SD (n=6 independent biological replicates). **P* < 0.05, ** *P* < 0.01, *** *P* < 0.001, and *****P* < 0.0001.


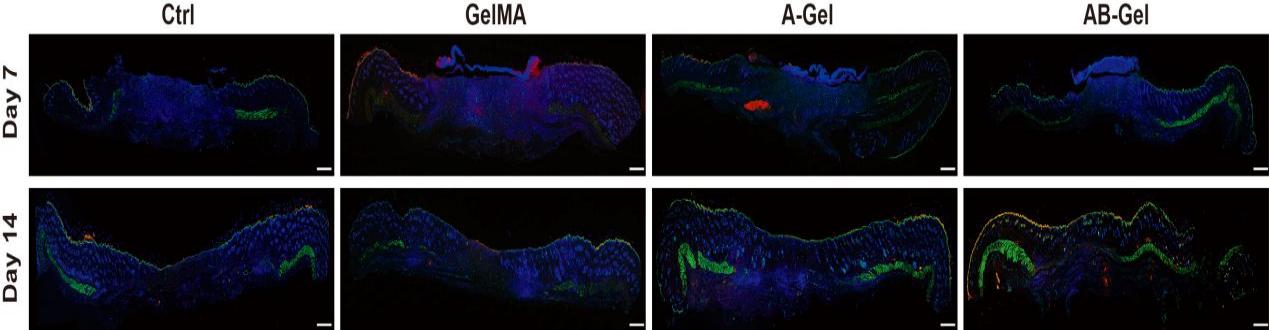


**Figure. S9. Immunofluorescent images of CD31 (green) /LYVE-1 (red) staining in the wound edge region of the AB-Gel group in 7-day and 14-day skin specimens.** Scale bar: 200 μm**.**


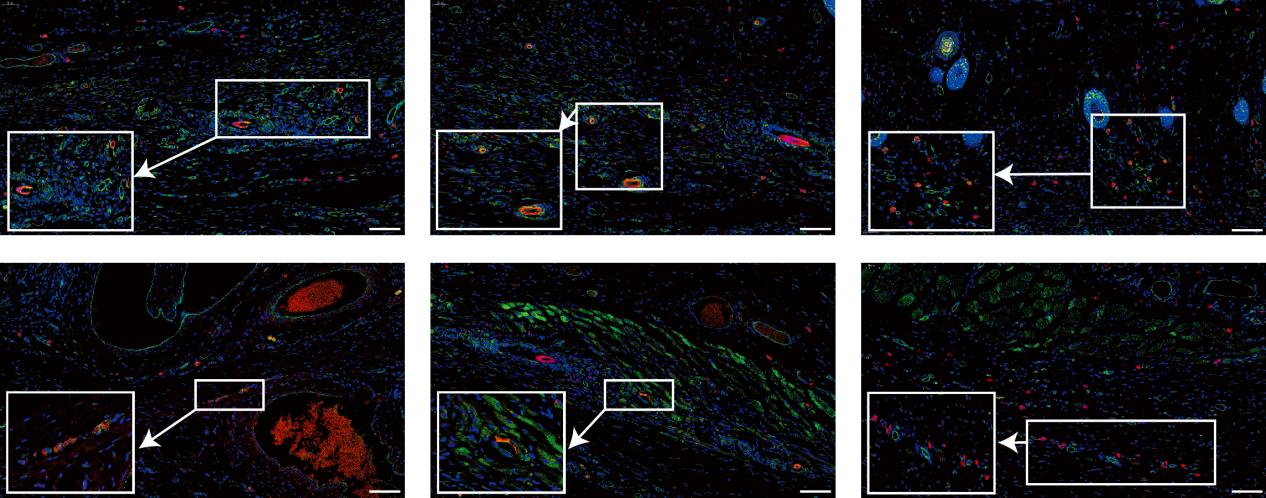


**Figure. S10. Immunofluorescent images of CD31 (green) /LYVE-1 (red) staining in the wound edge region of the AB-Gel group in 14-day skin specimens.** Scale bar: 100 μm**.**


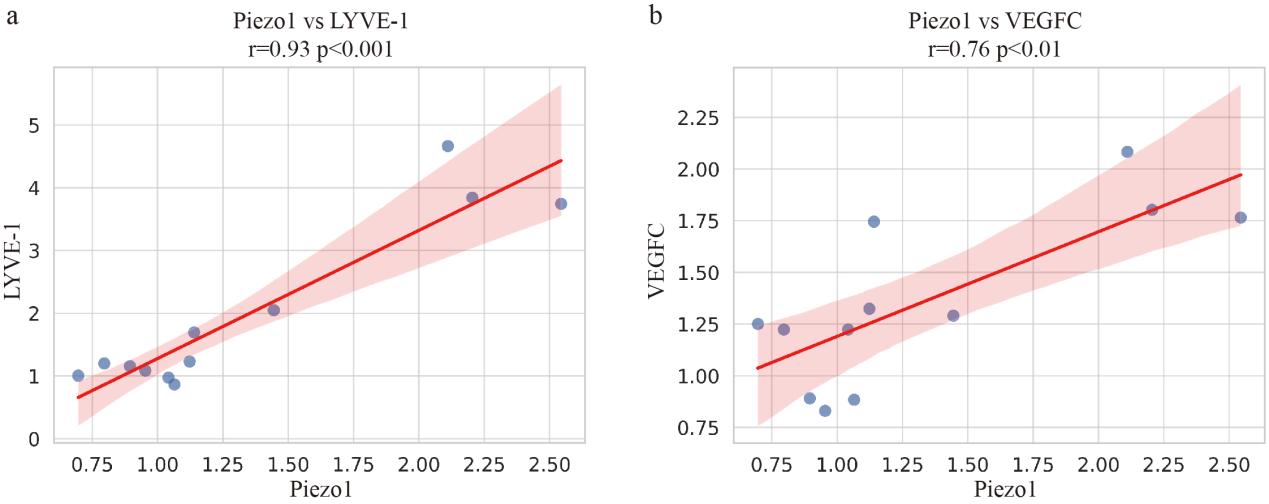


**Figure. S11. Correlation analysis of Piezo1 with LYVE-1 and VEGFC.** Scatter plots showing the correlations between Piezo1 and LYVE-1 (a) and VEGFC (b) Linear regression lines with 95% confidence intervals are presented. Pearson’s correlation coefficients (r) and significance levels are indicated in each panel. Piezo1 expression exhibited a strong positive correlation with LYVE-1 (r = 0.93, p < 0.001) and a moderate positive correlation with VEGFC (r = 0.76, p < 0.01), suggesting a potential promoting role of Piezo1 in lymphangiogenesis. (n=12 independent biological replicates).


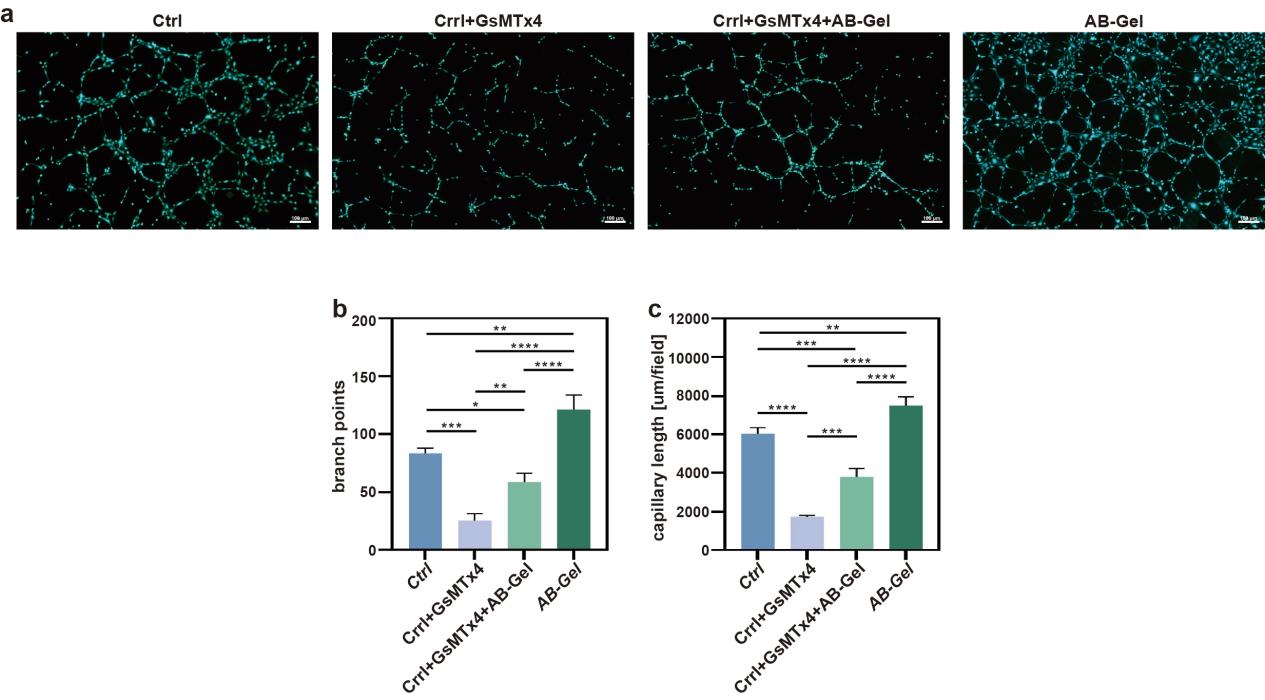


**Figure. S12. Piezo1 inhibition/rescue assay during HLEC tube formation.** (a) Representative images and quantification of HLEC tubular networks under Ctrl, Ctrl + GsMTx4, Ctrl + GsMTx4 + AB-Gel, and Ctrl + AB-Gel groups. (b and c) Quantitative analysis of lymphatic fluorescence expression. (n = 3 independent biological replicates). **P* < 0.05, ** *P* < 0.01, *** *P* < 0.001, and *****P* < 0.0001.

**Table S1. The gene-specific primer sequences**

**
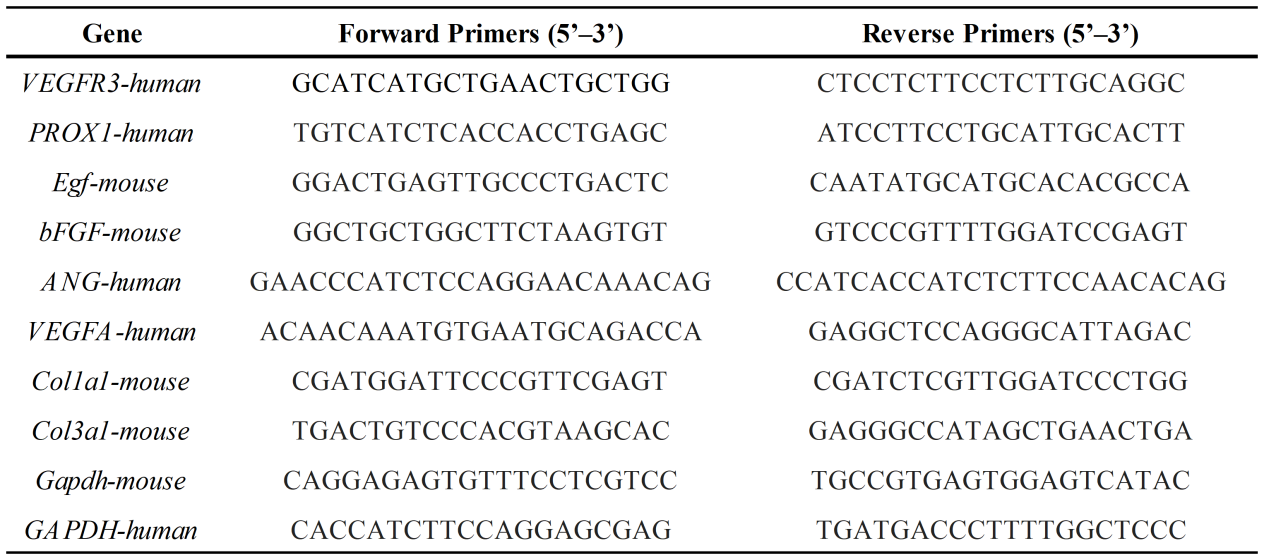
**
